# A careful reassessment of anthracycline use in curable breast cancer

**DOI:** 10.1038/s41523-021-00342-5

**Published:** 2021-10-08

**Authors:** Sara Alsterlind Hurvitz, Nicholas P. McAndrew, Aditya Bardia, Michael F. Press, Mark Pegram, John P. Crown, Peter A. Fasching, Bent Ejlertsen, Eric H. Yang, John A. Glaspy, Dennis J. Slamon

**Affiliations:** 1 Department of Medicine, David Geffen School of Medicine, University of California, Los Angeles, Jonsson Comprehensive Cancer Center, Los Angeles, CA USA; 2 Massachusetts General Hospital, Harvard Medical School, Boston, MA USA; 3 University of Southern California, Los Angeles, CA USA; 4 Stanford Comprehensive Cancer Institute, Palo Alto, CA USA; 5 Department of Medical Oncology, St. Vincent’s University Hospital, Dublin, Ireland; 6 Department of Gynecology and Obstetrics, University Hospital Erlangen, Friedrich-Alexander University Erlangen-Nuremberg, Comprehensive Cancer Center Erlangen-EMN, Erlangen, Germany; 7 Department of Oncology, Rigshospitalet, Copenhagen University Hospital, Copenhagen, Denmark

**Keywords:** Breast cancer, Chemotherapy

## Abstract

It has been over three decades since anthracyclines took their place as the standard chemotherapy backbone for breast cancer in the curative setting. Though the efficacy of anthracycline chemotherapy is not debatable, potentially life-threatening and long-term risks accompany this class of agents, leading some to question their widespread use, especially when newer agents with improved therapeutic indices have become available. Critically assessing when to incorporate an anthracycline is made more relevant in an era where molecular classification is enabling not only the development of biologically targeted therapeutics but also is improving the ability to better select those who would benefit from cytotoxic agents. This comprehensive analysis will present the problem of overtreatment in early-stage breast cancer, review evidence supporting the use of anthracyclines in the pre-taxane era, analyze comparative trials evaluating taxanes with or without anthracyclines in biologically unselected and selected patient populations, and explore published work aimed at defining anthracycline-sensitive tumor types.

## Introduction: The unavoidable problem of overtreatment in early-stage disease

“As to diseases, make a habit of two things—to help, or at least to do no harm” Hippocrates

Since the first publications of cytotoxic chemotherapy for early-stage breast cancer well over five decades ago^1,2^, numerous chemotherapeutic agents and regimens have been developed and tested with varying success for improvement in long-term outcomes. Compelling data from multiple studies including meta-analyses have concluded that adjuvant systemic chemotherapy reduces the risk of metastatic recurrence and improves overall survival (OS)^3^. However, it is clear that not all patients require chemotherapy to become and remain cancer free. Although risk stratification of patients has become better using more sophisticated assessment of clinical risk, including subtyping of breast cancer and more recent use of genomic assays, there is still no completely accurate way to distinguish those patients who are rendered truly “disease free” by local measures vs those who have microscopic metastases and could benefit from cytotoxic therapy. This challenge has led to the overtreatment of many women.

Over 90% of breast cancer is localized to the breast and regional lymph nodes (LNs) at diagnosis^4^. Random assignment trials utilizing surgery alone as a control arm demonstrated that 75% of patients with negative axillary nodes are recurrence free at 10 years without systemic therapy^5^. A large meta-analysis of trials comparing adjuvant chemotherapy to no chemotherapy including all breast cancer subtypes, indicated that over 50% of patients will be free of cancer recurrence at 10 years in the absence of chemotherapy^3^. Although hormone receptor-negative (HR−) breast cancer is considered to be more aggressive, up to two-thirds of patients with HR− cancer confined to the breast will be cancer free at 16 years without any systemic therapy^6^. Moreover, while the benefits of chemotherapy in patients with regional LN involvement has been consistently demonstrated, roughly one-quarter of these patients are disease free at 10 years with surgery alone^5,7^. In order to abide by the primary guiding principle of medical ethics—avoiding harm to the patient—clinicians are charged with the daunting task of not only identifying patients most likely to benefit from chemotherapy but also utilizing the systemic regimen that has the highest short-term therapeutic index possible combined with the minimum risk of long-term, significant, treatment-related toxicities. This goal is made all the more challenging to achieve as physicians and patients, alike, want to avoid a recurrence and thus will often err on the side of overtreatment.

Advances in molecular characterization and biological subtyping have provided the opportunity to develop more targeted and effective, less toxic interventions for several breast cancer subgroups. In the early days of adjuvant systemic therapy, clinical trials exercised no molecular selection. Thus, patients with HR-negative breast cancer were included in endocrine therapy studies^8–12^. These trials were generally positive, owing to the fact that the majority of breast cancers express HR, but we now know that patients with HR-negative disease do not benefit significantly from the addition of endocrine therapy^13^. The molecular classification of breast cancer is also facilitating new interpretations of results generated from randomized studies of systemic chemotherapy in which all subtypes of breast cancer were included without molecular selection. One such observation, based on the analysis of multiple studies comparing anthracycline to non-anthracycline regimens, is that the incremental benefit imparted by anthracyclines is likely restricted to a small subset of breast cancer.

In this review, we conduct a comprehensive analysis of the published literature relating to anthracyclines in early-stage breast cancer in order to define an evidence-based approach to the use of these agents in the curative setting.

## The benefit of anthracyclines in unselected patients in the era prior to molecular subclassification

Over a half-century has passed since Fisher and colleagues first evaluated perioperative cytotoxic chemotherapy for breast cancer in an attempt to reduce the risk of metastatic recurrence after radical mastectomy^1^. While the study was considered negative, multiple subsequent trials were conducted to evaluate systemic agents and regimens in the adjuvant setting. In several of these, significant improvements in relapse-free survival (RFS) and OS were observed^14^. Beginning in the 1970s, Bonadonna and colleagues evaluated the CMF regimen (cyclophosphamide, methotrexate, 5′-fluorouracil) in a series of clinical trials^15^. With over two decades of median follow up, these studies definitively demonstrated significant increases in RFS and OS with CMF compared to surgery alone.

Shortly after the FDA’s approval of doxorubicin in 1974^16^, Stephen Jones and colleagues published the results of their trial evaluating the novel combination of doxorubicin and cyclophosphamide (AC) for metastatic breast cancer (MBC)^17^, based on Jones’ preclinical demonstration of synergy between these two agents over a wide range of doses and schedules in vitro and in vivo^18^. Impressive responses were noted, with 80% experiencing an objective response, including six complete responses (CR). While this regimen was clearly active, two patients experienced congestive heart failure (CHF) after receiving a cumulative dose of 550 mg/m^2^ of doxorubicin, and one patient developed ST-T wave changes after a cumulative dose of 265 mg/m^2^, leading the authors to question whether an alternative schedule or dosing regimen would improve safety.

Subsequent to the Jones’^17^ publication, a number of clinical trials were conducted to compare anthracycline-based regimens to non-anthracycline regimens and suggested anthracyclines may improve outcomes in the metastatic setting^19–21^. Based on these results, in the early 1980s a prospective randomized study in locally advanced, nonmetastatic breast cancer was initiated to evaluate postoperative CMF plus vincristine and prednisone (CMFVP) for 12 months vs CAF (cumulative dose of 300 mg/m^2^ doxorubicin) for 6 months followed by CMFVP for 6 months^22^. In this small study (*N* = 41), DFS trended toward improvement in anthracycline-treated patients (*p* = 0.05). Since then, at least 20 randomized studies^9,23–34^ (Supplementary Table 1A, B) have compared anthracycline to non-anthracycline-based adjuvant therapies. Of these, 13 studies which collectively enrolled 12,075 participants showed no benefit with the use of an anthracycline (Supplementary Table 1A)^9,23–34^, three^23,35,36^ (*N* = 1883) showed a benefit in DFS but not in OS and four^37–40^ (*N* = 4859) showed a benefit in both DFS and OS with the use of an anthracycline (Supplementary Table 1B). These studies differed in their inclusion of LN+ or LN− disease, the inclusion of HR+ or HR− breast cancer, allowance of endocrine therapy, and inclusion of premenopausal and postmenopausal patients. Importantly, these studies were conducted prior to the routine use of HER2 testing, thus the patient population was mixed and no one with HER2-positive (HER2+) cancer received trastuzumab. As will be discussed later, this latter point is critical because *HER2*- amplified cancers that have co-amplification of the topoisomerase 2 gene locus—*TOP2A*, may be uniquely sensitive to anthracyclines.

In 2005, the EBCTCG reported a meta-analysis from randomized trials begun prior to 1996 of systemic therapy (chemotherapy, endocrine therapy, or chemoendocrine therapy) for early breast cancer^41^. Over 14,000 women were included in studies comparing anthracycline-containing regimens to CMF-based regimens. This meta-analysis demonstrated a statistically significant 0.8% per year difference in recurrence rate and a 3.1% absolute improvement in 10-year mortality associated with anthracycline-based therapy. These data indicated that anthracyclines were associated with an 11% improvement in relative risk of recurrence and a 16% improvement in relative risk of death compared to CMF-based therapy. In 2012, this meta-analysis was updated^3^ and showed that compared to standard CMF, four cycles of standard AC chemotherapy (*N* = 5122) was not associated with a significant difference in 10-year risk of recurrence (42.1% CMF vs 41.0% AC, *p* = 0.76) nor improvement in breast cancer mortality (32.5% CMF vs 31.6% AC, *p* = 0.67) (*N* = 5000). A small but significant benefit was seen with the use of higher cumulative anthracycline dosage compared to CMF (*N* = 9527) in terms of recurrence (CMF 33.8% vs 31.2% anthracycline, *p* = 0.003) and breast cancer mortality (24.1% CMF vs 20% anthracycline, *p* = 0.00001). Again, HER2 status was neither available nor considered at the time of reporting these data.

In summary, some studies demonstrated that adjuvant anthracyclines were superior, but inconsistently, and then only slightly better than CMF. A meta-analysis was required to confirm this benefit. The ability to distinguish those patients who were receiving a large benefit from anthracyclines from those who received no benefit was obscured by the fact that none of the prospective studies stratified by HER2 status, several did not include endocrine therapy for HR+ disease and trastuzumab-based therapy was not available for patients with HER2+ disease at the time these studies were conducted.

## The advent of taxanes

A collaborative effort in the 1960s–70s by the National Cancer Institute with the US Department of Agriculture in which thousands of plants were screened for anticancer activity led to the discovery of the first taxane, paclitaxel, from the bark of the Pacific Yew, paving the way to the development of docetaxel, a semi-synthetic analog from the renewable and more readily available leaves of the European yew tree^42–44^. Both drugs were shown to be highly active in MBC. Chan et al.^45^ demonstrated that docetaxel produced a significantly higher objective response rate than doxorubicin in a randomized trial in metastatic disease, the only agent shown to be superior to anthracycline in this setting. In this study, doxorubicin was associated with a 5% incidence of cardiac death.

This prominent activity of the taxanes in metastatic disease prompted early and extensive study of these drugs in the adjuvant setting. In contrast to the findings from many individual randomized studies of anthracycline-based therapy that showed no advantage in DFS or OS compared to CMF, the majority of individually randomized trials evaluating the use of taxanes in the curative setting were positive^46–51^, though there were a few exceptions^52,53^. Unlike the situation with anthracyclines, a meta-analysis was not needed to demonstrate the benefit of taxanes in the adjuvant treatment of breast cancer. Nevertheless, several meta-analyses were conducted, confirming that the addition of a taxane to anthracycline-based therapy significantly reduced the risk of recurrence and death^3,54,55^. Unfortunately, the lack of a non-anthracycline, taxane-based control arm in these early studies prevented the critical assessment of whether anthracyclines added any benefit to a taxane-based regimen.

Had taxanes been developed before anthracyclines, it is likely that prospective comparative trials would have been required to demonstrate that adding an anthracycline, with its risk of life-threatening toxicity, provides a substantial benefit in terms of DFS and OS compared to a non-anthracycline, taxane-based regimen. Instead, taxanes were developed subsequent to anthracyclines and as such all early studies compared anthracycline-based regimens to taxane plus anthracycline regimens. In fact, 20 years passed from the publication of the first clinical trial publication of paclitaxel^56^ to the first publication of a head-to-head clinical comparison of a taxane to an anthracycline for early-stage disease^57^.

### Head-to-head comparisons of anthracycline vs taxane in the curative setting

In 1997, Valero and colleagues published promising activity from a phase I study of a new “TC” regimen comprised of docetaxel (75 mg/m^2^) combined with cyclophosphamide (600 mg/m^2^)^58^. Stephen Jones, lead author of the original trial of AC for breast cancer in 1975^17^ was the first to conduct a formal head-to-head comparison of four cycles of AC vs TC^57^ in 1016 patients with stage I–III breast cancer. Approximately two-thirds of patients had HR+ disease (all of whom received endocrine therapy) and over 50% of patients had LN involvement (majority <4). With 5 years follow up, the DFS was significantly higher in patients treated with TC (86 vs 80%, *p* = 0.015)^57^. This improvement in DFS was seen regardless of age, LN status, or HR status. With 7 years of follow up, OS was significantly improved in patients treated with TC (87 vs 82%, *p* = 0.032)^59^ regardless of age or HR status. There were four deaths in the AC arm likely related to treatment; one due to CHF and three others due to myelodysplasia (MDS), myelofibrosis, and acute myeloid leukemia (AML). There were no such deaths reported in the TC arm.

A second adjuvant trial, conducted by the Alliance, directly compared a taxane to an anthracycline^60^. In contrast to the US Oncology study^57^, this study utilized single-agent paclitaxel and compared it to the anthracycline doublet, AC, and was a non-inferiority design^60^. A 2 × 2 design was utilized to also compare shorter vs longer therapy (four vs six cycles of AC and 12 vs 18 weeks of paclitaxel). This trial, which was open from 2002–2008, originally aimed to enroll 4646 patients based on 89% power and 567 RFS events. However, it closed after enrolling 3871 patients. In addition, several changes to the protocol design, including changes to eligibility and treatment regimens, occurred during the study. The original study design utilized standard q3 weekly AC and q-weekly paclitaxel (80 mg/m^2^). However, in 2003, after 571 patients had been enrolled, both regimens changed to q2 weeks (with paclitaxel dose increased to 175 mg/m^2^). Adjuvant trastuzumab was not incorporated until after 2005. At a median 6.1 years follow up and 437 RFS events (23% fewer events than required for 89% power), non-inferiority of paclitaxel could not be concluded with a HR of 1.26 favoring AC. Five-year RFS in AC vs paclitaxel arms was 91 and 88%, respectively. The authors acknowledged the limitations of this study relating to accrual and multiple changes in study protocol, but were reassured by post hoc statistical analyses indicating the overall results would not have been different had full accrual been met, or study design not changed. Fewer than half the patients had HER2 status available and of these, 16% were HER2+. The multivariable analysis did not include HER2 status. The fact that taxane-treated patients did not receive cyclophosphamide is another potential limitation of this study. No difference in 5-year OS was observed (95% AC vs 94% T), in contrast to the Jones study, with the noted major caveat that the Jones study had added cyclophosphamide to the taxane. Importantly, seven patients, all in the AC arm, developed treatment-related AML or MDS, all of whom died.

In addition to the two above adjuvant studies, a phase III neoadjuvant study from the National Cancer Center in Korea directly compared anthracycline to taxane-based therapy. A total of 204 patients with stage II–III breast cancer (all subtypes) were randomized to receive four cycles of q3 weekly docetaxel plus capecitabine (TX) or AC^61^. After surgery, all patients crossed over to receive four cycles of the other treatment. Pathologic complete response (pCR) in the breast was significantly better in the TX arm (21 vs 10%, *P* = 0.024). No difference in DFS was seen, an expected finding given the crossover design of the study.

Results from a substudy of the phase III EORTC 10041/BIG 03-04 (MINDACT) trial has also questioned whether anthracycline-based regimens are needed in the taxane era^62^. In this analysis, 1301 patients with T1–T3 operable tumors and up to three positive nodes were randomized 1:1 to standard anthracycline therapy (70% anthracycline without taxane, 30% anthracycline plus taxane) or TX. Patients with HER2+ tumors received standard trastuzumab. Though underpowered to determine superiority for TX, the 5-year DFS was similar for the anthracycline arm (88.8%) and DX arm (90.7%) (HR, 0.83; *p* = 0.26) as was the 5-year OS (96.2% anthracycline vs 96.3% DX, HR 0.91, *p* = 0.72).

### Studies comparing taxane/anthracycline vs taxane (in a mixed population of HER2+ and HER2− disease)

One of the earliest trials to be published comparing an anthracycline plus taxane to a taxane without an anthracycline was the JCOG9802 trial in which all subtypes of MBC were prospectively randomized to receive six cycles of AC, docetaxel, or alternating treatment with AC and D (three cycles of AC followed by three cycles of D)^63^. Patients were eligible if they had taxane naïve, HR− or endocrine-resistant HR+ breast cancer. Prior adjuvant anthracycline use was allowed. A total of 441 patients were accrued to this multicenter study. Approximately 85% of patients were anthracycline-naïve. Time to treatment failure (TTF), the primary endpoint, was similar in each of the three arms (6.4 mos AC, 6.4 mos D, 6.7 mos AC-D). OS was 22.4 months in the AC arm, which tended to be worse compared to the D-arms (25.7 mos in D arm, *p* = 0.09 and 25.0 mos in AC-D arm, *p* = 0.08). Responses were observed in 29% of patients treated with AC, 40% treated with D, and 35% treated with ACD. Tumors were not tested for HER2 and trastuzumab was not used. Moreover, the dose of both the anthracycline (40 mg/m^2^) and taxane (60 mg/m^2^) used in this trial were somewhat lower than used in the adjuvant setting, possibly impacting results. That said, these data did not suggest any incremental benefit derived from adding an anthracycline to docetaxel in the metastatic setting.

A phase III randomized trial (N-SAS BC 02) conducted at 84 centers in Japan evaluated whether single-agent taxane has a non-inferior DFS compared to anthracycline/taxane-based adjuvant therapy for LN+ breast cancer^64^. From 2000–2006, 1060 patients were randomly assigned to receive every 3 weeks either four cycles of AC followed by four cycles of paclitaxel (175 mg/m^2^) (ACP), four cycles of AC followed by four cycles of docetaxel (ACD), eight cycles of paclitaxel (P), or eight cycles of docetaxel (D). A 2 × 2 factorial design was utilized to compare AC-taxane-containing regimens (ACP and ACD) to taxane without anthracycline (P and D) and to compare paclitaxel-containing regimens (ACP and P) to docetaxel containing regimens (ACD and D). With a median follow up of 7 years, non-inferiority of the single-agent taxane could not be demonstrated (HR 1.19; 90.3% CI, 1.012–1.405, *p* = 0.30). This finding may have been influenced by the use of q3 weekly P in the two arms which was clearly inferior to q3 weekly D-containing regimens (DFS HR, 0.72; *p* = 0.0008 and OS HR 0.75; *p* = 0.035). Of all four arms, the median DFS was numerically longest in the D arm (ACP: 84.4 mos, ACD: 85.7 months, D: 87.9 mos, and P: 78.0 mos). Interestingly, patients with HER2+ breast cancer (16% of population, none treated with trastuzumab) did appear to derive greater benefit from the addition of anthracycline to P (HER2+: ACP vs P [HR, 1.85; 95% CI, 1.15–2.98]).

## Does the *HER2* alteration affect sensitivity to anthracyclines?

When evidence emerged indicating that HER2 amplification is a poor prognostic indicator in breast cancer^65–69^, investigators began to query whether this outcome may be linked to altered sensitivity to various standard chemotherapies. To this end, archived tumor samples from a number of adjuvant trials conducted in the pre-trastuzumab era were analyzed. Initially, several studies evaluating non-anthracycline-based chemotherapy indicated that HER2 overexpression was associated with resistance to chemotherapy and thus may, at least in part, explain the poor outcome associated with this genetic alteration^70–72^.

Subsequently, a number of investigators evaluated whether the HER2 alteration affects sensitivity to anthracycline-based therapy, using a variety of laboratory methods to evaluate HER2 expression and amplification status. Tumor samples were retrospectively assessed for HER2 status from a total of 12 separate randomized trials^73–85^ comparing non-anthracycline to anthracycline-based adjuvant chemotherapy (Table 1). Of these, three demonstrated a significant interaction between HER2 status and treatment^73,76,83^, indicating that HER2+ breast cancer is associated with a greater benefit in terms of DFS and/or OS from anthracyclines than HER2− breast cancer. One of the earliest of these analyses was from the NSABP B11 trial in which patients with LN+, HR− disease were randomized to receive l-phenylalanine mustard plus 5-fluorouracil alone or in combination with doxorubicin^73^ Those with HER2 overexpression had significant DFS, RFS, and OS benefit with the addition of doxorubicin. In contrast, those with normal HER2 expression had similar outcomes regardless of anthracycline use. Another large study, the Canadian Mammary.5 (MA.5)^83^ also evaluated HER2 status retrospectively. In this trial, adjuvant CEF was compared to CMF in LN+ disease. Improvements in RFS and OS from anthracycline use were restricted to those patients with *HER2* amplified tumors. Subsequently, PAM-50 analysis was performed on these samples, indicating that the HER2-enriched subtype benefited significantly from CEF, however other subtypes, including basal-like breast cancers (frequently TNBC), did just as well with CMF^86^.

**Table 1:** Summary of studies anthracycline vs non-anthracycline chemo: outcome based on HER2.

Study (Year)	Ref	Regimens	Dz setting	*N*	Results	Does HER2 predict benefit from anthracycline?	HER2 test assay/definition
NSABP B11^a,b^ 1998 Paik et al	73	PAF PF	ADJ	638 >90% tested	DFS HER2+ RR = 0.60 HER2− RR = 0.96; *p* int = 0.02 OS HER2+ RR = 0.66 HER2− RR = 0.90; *p* int = 0.15	Yes significant (DFS) Non-significant trend (OS)	IHC “any tumor cell showed definite membrane staining resulting in fish-net appearance” used cocktail technique with mAb-1 and pAb-1 antibodies
NSABP B15^a,b^ 2000 Paik et al	74	AC vs AC-CMF vs CMF	ADJ	1355	DFS HER2+ RR = 0.84 HER2− RR = 1.02; *p* int = 0.19 OS HER2+ RR = 0.82 HER2− RR = 1.07; *p* int = 0.11	Maybe non-significant trend (DFS and OS)	cocktail of TAB-250 and p-Ab
Czech^c^ 2000 Petruzelka et al	75	AC vs CMF	ADJ	62	HR for HER2+ and HER2− not reported but *p* interaction not significant	No	IHC CB11
GUN-3^c,d^ 2001 De Laurentiis et al	76	CMF vs CMF/EV	ADJ	123	OS: HR HER2+ = 0.85; HR HER2− = 1.64; *p* int = 0.05	Yes significant for OS	IHC using MAb-1 Ab
Belgian^b,d^ Di Leo et al 2001	77	CMF vs EC (E: 60 mg/m^2^) vs HEC (E: 100 mg/m^2^)	ADJ	481	EFS HEC vs CMF: HER2+ (by CB11 and 4D5): HR = 0.33 HER2 neg: HR = 1.16; *p* int = 0.10. EFS EC vs CMF: HER2+ (by CB11 and 4D5): HR = 0.86 HER2-neg: HR = 1.24; *p* int = 0.21	Maybe non-significant trend toward better EFS with anthracycline, especially high dose in HER2+	IHC CB11 and 4D5 and cocktail of TAB-250 and p-Ab HER2+ if >1% cells stained -High (8/21, 38%) false positive with cocktail
Belgian^b,d^ Di Leo et al 2002	78	CMF vs EC (E: 60 mg/m^2^) vs HEC (E: 100 mg/m^2^)	ADJ	354	EFS HEC vs CMF HER2+ HR = 0.70 HER2− HR = 1.19; *p* int = 0.57 EFS EC vs CMF HER2+ HR = 0.61 HER2− HR = 1.52; *p* int = 0.18	Maybe non-significant trend toward better EFS with anthracycline, especially high dose in HER2+	FISH ratio >2
Milan^a,b^ 2003 Moliterni et al	79	CMF vs CMF-A	ADJ	506	DFS trend in favor of A in HER2+ only HER2+ HR = 0.83 HER2− HR = 0.1.22 *p* interaction = 0.251 OS in favor of A in HER2+ only HER2+ HR = 0.61 HER2− HR = 1.26 *p* interaction = 0.052	Maybe almost Significant	IHC CB11 Ab positive if “strong membrane labeling”
Spanish^c^ 1999 Vera et al	80	FAC vs CMF	ADJ	141	5-year OS HER2+ 72% FAC vs 42% CMF HER2− 82% FAC vs 84% CMF	Maybe NR whether significant	IHC CB11 Positive if >50%
DBCG 89D^a,b,d,e^ 2005 Knoop et al	81	CEF vs CMF	ADJ	805	EFS: HR = 0.747 HER2+ HR = 0.789 HER2−; *p* int = 0.81 OS: HR = 0.725 HER2+ HR = 0.818 HER2−; p int = 0.63	No	IHC Dako Ab If IHC 2+ FISH ratio >2
GOIRC^a,b^ 2005 Colozza et al	82	CMF vs E	ADJ	266	8-year EFS HER2+: E 66% CMF 70%* HER2−: E 60% CMF 69%; *p* int = 0.6628 8-year OS HER2+: E 76% CMF 68%* HER2−: E 85% CMF 87%; *p* int = 0.311	Maybe (trend toward better OS if HER2+) * EFS HER2+ CMF is higher than OSHER2+ thus EFS unreliable	IHC CB11 >50% stained tumor cells and HercepTest
NCIC MA.5^a,b,d^ 2006 Pritchard et al	83	CEF vs CMF	ADJ	628	5-year DFS HER2+: HR = 0.52 HER2−: HR = 0.91; *p* int = 0.02 5-year OS HER2+: HR = 0.65 HER2−: HR = 0.1.06; *p* int = 0.07	Yes significant better DFS, trend toward better OS with anthracycline if HER2+	IHC, FISH, PCR 89% tumors tested FISH positive >2
BR9601 2008 Bartlett et al	84	CMF vs E-CMF	ADJ	303	RFS E-CMF vs CMF HER2+: HR = 0.910 HER2-: HR = 0.495; *p* int = 0.168 OS E-CMF vs CMF HER2+: HR = 1.046 HER2−: HR = 0.454; p int = 0.083	No non-significant trend toward a better outcome with anthracycline if HER2−	FISH >2 (used FISH for all analyses) IHC by HercepTest, 2+/3+ = positive used TMAs
BR9601/NEAT^d^ 2010 Bartlett	85	CMF vs E-CMF	ADJ	1762	RFS E-CMF vs CMF HER2+: HR = 0.77 HER2−: HR = 0.79; *p* int = 0.85 OS E-CMF vs CMF HER2+: HR = 0.74 HER2−: HR = 0.84; *p* int = 0.55	Maybe non-significant trend toward OS with anthracycline if HER2+	FISH >2.0 = HER2 amplified Chrom 17 duplication >1.86 CEP signals per cell

*A* doxorubicin, *ADJ* adjuvant, *C* cyclophosphamide, *DFS* disease free survival, *E* epirubicin, *F* fluorouracil, *GOIRC* Gruppo Oncologico Italiano di Ricerca Clinica, *HR* hazard ratio, *M* methotrexate, *MBC* metastatic breast cancer, *NAC* neoadjuvant chemotherapy, *NR* not reported, *P* melphalan, *pac* paclitaxel, *RFS* relapse-free survival,*T* docetaxel, *Tam* tamoxifen, *V* vincristine.

^a^Included in Gennari pooled analysis Table 2C.

^b^Included in Dhesy-Thind pooled analysis Table 2C.

^c^Only available in abstract form and data also gathered from Dhesy-Thind and Pritchard.

^d^Included in Di Leo meta-analysis Table 2C.

^e^Also refer to Table 3 (topoisomerase IIa analysis reported).

Analysis of another nine trials demonstrated that HER2-positive breast cancers tended to have greater benefit from anthracyclines (in either DFS or OS outcomes) compared to HER2− breast cancers. However, these trends did not reach a statistically significant level of interaction for either outcome measure (Table 1)^74,77–80,82,85^. Paik and colleagues analyzed HER2 expression on tumor samples from 1355 of 2194 (62%) patients enrolled on the NSABP B15 study in which patients with LN+ breast cancer received adjuvant therapy with either AC, CMF, or AC→CMF^74^. Outcomes tended to be better in patients with HER2 overexpressing breast cancer treated with AC-based therapy (DFS for HER2+, RR = 0.84, OS for HER2+, RR = 0.82), though this also did not reach statistical significance. In contrast, patients with HER2− breast cancers had virtually overlapping outcomes with anthracycline vs non-anthracycline-based therapy, with a slight trend toward better outcomes with CMF treatment (DFS HER2− RR = 1.02; OSHER2− RR = 1.07). Similarly, HER2 analysis (*N* = 506) from a study in Milan^79^ demonstrated a strong trend toward survival benefit with anthracyclines only in those with HER2-overexpressing disease (OS CMF-A vs CMF HER2+: HR = 0.61, HER2− HR:1.26; *p* interaction = 0.052). Likewise, analysis of samples (*N* = 481) from a Belgian study^77^ comparing CMF to high dose EC (HEC, epirubicin at 100 mg/m^2^) indicated a strong trend toward improved event-free survival (EFS) for those patients with HER2 overexpressing breast cancer (HR: 0.33) whereas those with HER2− breast cancer seemed to derive just as much benefit from CMF (HR:1.16). While analysis from the BR9601 study (*N* = 303)^84^ showed no significant interaction between treatment, HER2 status, and outcome, samples were tested from tissue microarrays (TMA) which, according to one study, may alter the reliability of HER2 result^87^. When the BR9601 samples were combined with samples from the similarly designed NEAT trial^85^, the sample size increased to 1762, and again a trend toward improved OS was noted in favor of anthracycline-based therapy for HER2+ (HR = 0.74) compared to HER2− disease (HR = 0.84; total HR 0.81; *p* = 0.02; *p* interaction = 0.55).

DBCG 89d, which evaluated samples from 805 patients (67%) randomized to CEF vs CMF, showed no difference in EFS when comparing hazard ratios for HER2+ and HER2− disease^81^. However, a recently published analysis^88^ of 686 samples from this study using the Prosigna Prognostic Gene Signature indicates that patients with the HER2-enriched subtype have a better distant relapse and OS when treated with CEF vs CMF. The same was not observed in the other intrinsic subtypes. Discordance between HER2-enriched status and *HER2* FISH status was noted. Of 217 patients with HER2-enriched tumors, 32 (15%) were *HER2−* by FISH. Only those that were HER2-enriched appeared to benefit from epirubicin. Of 469 classified as non-HER2-enriched, 38 (8%) were *HER2-*amplified by FISH but did not show benefit with epirubicin, though the sample size was quite small.

Given these somewhat conflicting results, from trials using different methods to define HER2 status, and some trials lacking sufficient statistical power to critically analyze efficacy endpoints in the defined HER2+ subset, Gennari and colleagues performed a pooled analysis of the interaction between HER2 and anthracycline benefit^89^. Eight studies (Table 2) including over 5000 patients comparing adjuvant anthracycline to non-anthracycline chemo were analyzed. Methods for defining *HER2* status differed across studies, as indicated above and in Table 1, and included IHC, FISH, and polymerase chain reaction. In spite of this heterogeneity, this meta-analysis, with its greatly increased power, clearly demonstrated that anthracycline benefit was restricted to those patients with HER2+ disease. HER2+ breast cancers had a 29% relative risk reduction for DFS events and 27% relative improvement in survival with the use of anthracycline. Conversely, there was no incremental benefit from an anthracycline vs a non-anthracycline regimen in HER2- disease. The interaction for both DFS and OS was statistically significant (*p* < 0.001 for both). It is notable that when HER2+ and HER2− breast cancers were analyzed together as a single group, the combined DFS (HR 0.90) and OS (HR 0.91) were better with the anthracycline regimens. However, in sensitivity analysis, this benefit is completely lost when the HER2+ breast cancers are removed from the meta-analysis. This relative benefit seen in the combined population is strikingly similar to that reported from the EBCTCG meta-analysis^3,41^ in which a slight improvement in DFS and OS was seen with the use of anthracyclines in a mixed pool of breast cancer patients unselected for HER2 status. Subsequently, a pooled analysis^90^ of these studies plus one other study^76^ confirmed the findings of the Gennari analysis (Table 2).

**Table 2:** Summary of meta-analyses evaluating the relationship of HER2 alteration on outcome with anthracycline vs non-anthracycline.

Study (Year)	Ref	Studies Included	Dz Setting	*N*	Outcomes	Notes regarding testing
Gennari 2008 J Natl Cancer Inst	89	NSABP B11 NSABP B15 GUN3 (OS only) Belgian (DFS only) Milan DBCG 89D GOIRC (OS only-methodologic issues w/ DFS) MA-5	ADJ	4286 DFS 4321 OS	DFS anthracycline vs non-anthracycline HER2+ HR = 0.71 HER2− HR = 1.00; *p* int <0.001 OS anthracycline vs non-anthracycline HER2+ HR = 0.73 HER2− HR = 1.03; *p* int <0.001	Pooled analysis No centralized reassessment of HER2 IHC and/or FISH used
Dhesy-Thind 2008 Breast Cancer Res Treat	90	MA-5 (*N* = 628) Milan (*N* = 506) NSABP-B11 (*N* = 638) NSABP-B15 (*N* = 1355) Belgian (DFS only) (included only EC and CMF arms and only results as tested by FISH, *N* = 237) DBCG 89d (*N* = 805) GUN-3 (OS only)^a^ (*N* = 123) GOIRC (OS only) (*N* = 266)	ADJ	4169 (DFS) 4321 (OS)	Significant benefit for anthracycline in HER2+: DFS HR = 0.71; (95% CI 0.60,0.83) OS HR = 0.73; (95% CI 0.62–0.86) No significant benefit of anthracyclines for HER2 negative	
Di Leo 2011 Lancet Oncol	87	Belgian MA-5 DBCG 89d NEAT BR9601	ADJ	3452	EFS anthracycline vs CMF HER2+ HR = 0.71 HER2− HR = 0·89; *p* int = 0.0485 OS anthracycline vs CMF: HER2+ HR = 0·73 HER2- HR = 0·91; *p* int = 0.0718	Tested all samples centrally Better concordance between external lab and national laboratories when whole tumor sections used rather than TMAs. Concordance 94% in a subset (*N* = 137) sampled at external and national lab HER2 FISH >2 = positive

*ADJ* adjuvant, *C* cyclophosphamide, *DFS* disease-free survival, *E* epirubicin, *F* fluorouracil, *FISH* fluorescent in situ hybridization, *HR* hazard ratio, *IHC* immunohistochemistry, *M* methotrexate, *MBC* metastatic breast cancer, *NR* not reported, *OS* overall survival, *pac* paclitaxel, *RFS* relapse-free survival, *TMA* tissue microarray.

In 2011, Di Leo and colleagues published another analysis of five studies using individual patient data (Table 2)^87^. Unique to this was the fact that the authors attempted to independently assess HER2 status in a centralized laboratory located in Tampere, Finland. However, when high rates of discordance were detected between the external laboratory in Tampere and the four national laboratories that tested the samples from each of the trials, it was decided that the use of TMA might be to blame and only a portion of patient samples (*N* = 137) from whole tumor sections were sent for repeat *HER2* FISH testing at the external central lab. The concordance rate for *HER2* results between the national laboratories and the external central laboratory for these samples was 94%. Four of the included studies^78,81,84,85^ had failed to demonstrate a significant association between *HER2* status and outcome with anthracycline-based therapy. However, when analyzed together with over 3400 samples and 1417 events, the EFS was significantly in favor of anthracyclines for HER2+ disease (HR = 0.71) compared to HR = 0.89 for HER2− breast cancer (*p* interaction = 0.0485). OS also trended toward an improved benefit with anthracyclines for HER2+ breast cancer (HR = 0.73) compared to HER2− (HR = 0.91).

A handful of other trials evaluated whether patients with HER2+ breast cancer receive greater benefit from dose intense anthracyclines, defined as either a higher cumulative dose or more frequent dosing in the curative setting (Table 3). One of the first of these analyses was conducted by Muss and colleagues and demonstrated a clear dose-response relationship with doxorubicin in patients with HER2+ cancers, but not those with HER2− disease^91–93^. In their initial analysis, samples from 397 patients treated in the CALGB 8541 study were analyzed for HER2 by IHC. In both the overall study and in the subset of patients included in this biomarker analysis, DFS and OS were improved in patients receiving higher doses of chemotherapy (groups 1 and 2). In those with HER2+ tumors, DFS and OS were significantly associated with a higher dose of chemotherapy. Focusing on all patients treated with high-dose chemotherapy, those with HER2+ tumors had a better DFS and OS compared to patients whose tumors were HER2−^91^. Tumor samples from an additional 595 patients were subsequently analyzed along with the original 397 samples^92^. In the group of 397 patients with a median follow up of 10.4 years, the association between HER2 and anthracycline dose was even stronger than reported in the initial analysis, though this dose-response relationship in HER2+ breast cancer did not appear to hold for doses of doxorubicin above 60 mg/m^2^. Indeed, similarly, an analysis of patients treated on the CALGB 9344 study^94^ in which patients were assigned to 60, 75, or 90 mg/m^2^ showed a comparable 5-year DFS irrespective of HER2 status.

**Table 3:** Summary of studies where all patients received anthracycline (compared different doses/frequency)—outcome based on HER2.

Study (Year)	Ref	Regimens	Dz Setting	HER2 evaluable *N*	Outcomes	Interaction HER2 and outcome	HER2 test assay/definition
CALGB 8541 8869^1^ Muss 1994 Thor 1998 Dressler 2005	91–93	FAC High dose (H) (HFAC) (A = 60 mg/m^2^ × 4 = 240 mg/m^2^ total) Standard (S) (FAC) (A = 40 mg/m2 × 6 = 240 mg/m^2^ total) Low dose (L) (LFAC) (30 mg/m^2^ × 4 = 120 mg/m^2^ total)	ADJ	992	5-year DFS H-S-L (based on IHC) HER2+ 71% - 52% - 50% HER2- 65% - 66% - 60% *p* interaction = 0.001 5-year OS H-S-L HER2+ 87% - 66% - 63% HER2- 77% - 82% - 78% *p* interaction <0.001	Yes Significant DFS and OS benefit with anthracycline in HER2+ only	IHC CB11 Positive >50% Dressler tested HER2 by IHC/FISH/PCR-all showed benefit of higher dose anthracyclines in HER2+
HE10/97 Kostopoulos 2006	99	E x 3→Tx 3→CMF x 3 E x 4→CMFx4	ADJ	394	OS *p* int = 0.73 DFS *p* int = 0.57	No	HercepTest 3+FISH >2
French series Petit 2001	97	FEC50 vs FEC100	NAC	79	Retrospective series Clinical response to six cycles Hi dose anthracycline and HER2+ predicted high objective response ORR HER2+ FEC50 12.5% FEC100 100% ORR HER2− FEC50 74% FEC100 70%	Maybe Non-significant trend toward ORR benefit with high dose anthracycline in HER2+ only	CB11 IHC 2+ or 3+
Belgian^1^ Di Leo 2001 and 2002	77, 78	EC (E: 60 mg/m^2^) vs HEC (E: 100 mg/m^2^) (vs CMF, see Table 2A)	ADJ	481 by IHC 236 FISH (117 HEC, 119 EC)	Predictive value of HER2 may vary according to the Abs used EFS HEC vs EC by IHC HER2+ (CB11/4D5): HR 0.58 HER2 neg: HR = 0.73; *p* int = 0.49 EFS HEC vs EC by FISH HER2+: HR = 0.90 HER2-: HR = 1.33, *p* int = 0.53	Maybe Non-significant trend toward better EFS with high dose anthracycline, in HER2+	IHC CB11 and 4D5 And cocktail of TAB-250 and p-Ab HER2+ if >1% cells stained-High (8/21, 38%) false positive with cocktail
FASG-05^2^ Arnould 2003	95	FEC50 vs FEC100	ADJ	332	10-year DFS HER2+ FEC100 55% FEC50 37% HER2- FEC100 39% FEC50 36% *p* int NR	Maybe Non-significant trend toward better DFS with high dose anthracycline in HER2+	IHC, Ab not stated IHC 2+ or 3+ on 0–3 scale
Dutch Rodenhuis 2006	100	FECx5 vs FECx4-HD chemo and ASCT Analysis of extra doses of anthracycline confounded by fact that FECx4 received high dose chemo/stem cell rescue	ADJ	801	621 HER2 neg-benefitted from less anthracycline and high dose chemo ASCT; HER2+ did not (resistant to alkylating agents); RFS FEC vs FEC-HD/ASCT HER2+ HR = 1.26 HER2− HR = 0.68 *p* interaction = 0.006	No Significant benefit with less anthracycline and HD chemo/ASCT if HER2 negative	IHC and CISH. IHC 3+ by 3B5 Ab Amplif >5 copies
GONO-MIG-1^1,2^ Del Mastro 2005	96	FEC21 v FEC14	ADJ	731	EFS FEC14 vs FEC21 HER2+ HR = 0.54 HER2− HR = 0.91; *p* int = 0.12 OS FEC14 vs FEC21 HER2+ HR = 0.59 HER2− HR = 0.79; *p* int = 0.38	Maybe Non-significant trend toward better EFS/OS with increased frequency anthracycline if HER2+	IHC CB11 IHC 3+ = positive (strong complete membrane staining >10% of tumor cells)
CALGB 9344 Hayes 2007	94	AC +/ pac (A at 60, 75, or 90 mg/m^2^) × 4	ADJ	1322	No anthracycline dose-response relationship with HER2: 5-year DFS by dose anthracycl. HER2+ 60 mg/m^2^ 63% 90 mg/m^2^: 63% HER2−: 60 mg/m^2^ 72% 90 mg/m^2^ 69%	No (taxane does better with HER2+)	IHC CB11 >50% staining = positive Or Herceptest 3+ Or FISH >2.0
Dhesy-Thind Meta-analysis 2007	90	Included CALGB 8541 (HFAC/FAC, *N* = 670), Belgian (HEC/EC by FISH, *N* = 236), GONO-MIG-1, *N* = 731)	ADJ	1637	HER2+ had significant DFS benefit with more intense anthracycline regimen HR = 0.54 (95% CI 0.38–0.79) HER2− no signif DFS benefit HR = 0.98		
ASG1–3 Fasching 2019	98	EC-Pac q3 weekly (E:90 mg/m^2^) vs E+Pac q2 weekly (E:120 mg/m^2^)	ADJ	906	HER2+ significant DFS benefit with ddET vs EC-P: HR = 0.24 (95% CI: 0.10–0.60) HER2− no significant benefit with dose-dense: HR = 1.45 (95% CI 0.86–2.45)	Yes	Not reported

Several additional studies^77,78,95–98^ also showed a trend toward a positive relationship between the HER2 alteration and benefit from dose intense anthracyclines. Dhesy-Thind^90^ performed a meta-analysis^78,92,96^, using FISH data from the Belgian study^78^ and data from the high dose and medium dose anthracycline arms from the CALGB 8541 analysis^91^. This showed that those with HER2+ breast cancer derive a significant DFS benefit from dose intense chemotherapy (HR = 0.54, 95% CI 0.38–0.79) whereas those with HER2− disease do not (HR = 0.98). While two studies^99,100^ failed to show an association with HER2 status and benefit from an additional cycle of anthracycline, these trials were confounded by the addition of either taxane^99^ or high dose chemotherapy followed by stem cell rescue^100^ in the arm with less anthracycline.

A meta-analysis conducted by the EBCTCG evaluated the benefits of giving chemotherapy drugs either more frequently or sequentially (instead of concurrently) in early-stage disease^101^. Dose intensification provided a modest benefit in recurrence risk for patients regardless of HER2 status. However, all arms of the included studies were treated with an anthracycline and the majority also received a taxane. Thus, this analysis was not designed to specifically address whether higher cumulative anthracycline doses are associated with benefit based on HER2 status and this analysis does not directly bear upon the question as to whether the addition of anthracyclines per se provides benefit.

Taken together, these data provided a credible link between the HER2 alteration and sensitivity to anthracycline-based therapy. Indeed, the first two FDA approvals for HER2 FISH testing were to risk stratify patients^102^ and select those who were at greater risk for recurrent disease^103^ and/or death and to select those who might benefit from anthracycline chemotherapy (https://fda.report/PMA/P980024S001)^104^. In spite of the circumstantial evidence supporting a link between the HER2 alteration and anthracycline efficacy, it was not clear whether HER2 was causal in heightened anthracycline sensitivity or if it was simply a surrogate marker for another molecular alteration that explained increased responsiveness to anthracyclines. To evaluate this, MCF7 cells were transfected with *HER2* cDNA to be rendered HER2 overexpressing and then treated with tamoxifen or chemotherapy. These cells proved resistant to tamoxifen both in vitro and in vivo, however, their sensitivity to anthracycline chemotherapy was unchanged despite HER2 overexpression^105^. Similarly, Pegram and colleagues transfected full-length human HER2/neu cDNA into MCF7, MDA-MB-231, MDA-MB-435, and BT-20 breast cancer cell lines and two ovarian cancer cell lines and compared the response of these cell lines in vitro and in vivo to that of mock-transfected parental lines^106^. These experiments also failed to demonstrate an association between HER2 overexpression and sensitivity or resistance to chemotherapy, including doxorubicin. Orr and colleagues transfected normal human mammary epithelial cells with *HER2* and determined sensitivity to multiple chemotherapy agents and also showed that HER2 overexpression is not associated with differential sensitivity to chemotherapy^107^. Further data was provided from Konecny et al. from an in vitro study analyzed at UCLA in which breast cancer samples from 140 chemotherapy-naïve patients from the University of Munich, Klinikum Grosshadern, taken at the time of surgery were treated with either CMF or FEC at different concentrations ex vivo^108^. This, too, showed no association between HER2 overexpression and resistance to either regimen. Before considering possible alternative reasons for differential anthracycline sensitivity being observed clinically in HER2+ disease, data relating to the benefits of adding anthracyclines to taxanes in HER2 negative disease will be explored.

## Prospective studies designed to evaluate taxane/anthracycline vs taxane only in HER2 negative early-stage disease

Although the above retrospective analyses did not consistently support the restricted use of anthracyclines to HER2+ breast cancers, the data clearly indicated that benefit from anthracyclines appeared to be limited, at best, in HER2− disease. Moreover, none of the above studies (Tables 1 and 2) involved the use of a taxane. Thus, a number of investigators aimed to prospectively evaluate whether the addition of anthracyclines to modern taxane-based regimens was associated with a meaningful benefit in patients with curable HER2− disease. In 2007, US Oncology Research initiated a randomized trial (USOR 06-090) designed to evaluate whether six cycles of docetaxel/doxorubicin/cyclophosphamide (TAC) is superior to six cycles of TC in LN positive or high-risk node-negative breast cancer. A second study, developed jointly by the NSABP and USOR was then initiated in 2009 to evaluate three arms: TCx6, TACx6, and TC plus bevacizumab x 6 with a planned enrollment of 3600 patients. The plan was to combine the TC and TAC arms from the USOR 06-090 and NSABP B-46-I/USOR 07132 studies. However, given the withdrawal of FDA approval of bevacizumab, it was decided to close this study after enrollment of only 1077 patients in early 2012. To convert the study to a non-inferiority design, a third study (NSABP B-49) was opened in 2012. In this trial, patients were randomized to TC or to four different anthracycline/taxane-based regimens. This study enrolled 1870 patients. The joint analysis of these three trials (collectively known as “ABC”) was published in 2017^109^ and marks the first to address whether a non-anthracycline, taxane-based regimen is non-inferior to the anthracycline/taxane regimens in terms of invasive DFS (iDFS). Of 4156 patients, 31% had TNBC, 59% had LN+ disease, and 51% had high-grade tumors. An interim analysis with a median follow up of 3.3 years and 399 observed iDFS events, demonstrated the observed HR was 1.23 (95% CI 1.01, 1.50; *p* = 0.04), thus non-inferiority could not be concluded. The absolute difference between treatment arms in 4-year iDFS was 2.5% (TC: 88.2%, TaxAC: 90.7%). Most of the benefit for the anthracycline arms was observed in those with four or more LN involved. In that group, the projected absolute difference between the two arms (by Kaplan–Meier analysis at 4 years) was 11% for TNBC and 5.8% for HR+. In the overall population, distant recurrences were observed in 5.3% of TC-patients vs 3.6% of anthracycline-patients. Deaths rates were similar in each arm (1.1% TC and 1.4% anthracycline). There were five acute leukemias diagnosed in anthracycline-treated patients, none in TC-treated patients. Notably, toxicity data was only published in the main manuscript for the NSABP B-49 trial. Safety data from the other two studies appear in the Data Supplement. No grade >3 cardiomyopathy was reported for TC however at least three patients died from cardiomyopathy or heart failure in the anthracycline arms, per the Supplementary Data. It is also notable that cardiac function was not measured throughout the study so occult cardiac dysfunction rates are not known.

Given the lack of centralized pathology review and the use of a HER2/CEP17 ratio of <2.2 (rather than the currently FDA approved <2.0), it is plausible that some patients with *HER2* amplified breast cancer may have been enrolled. Whether this is the case and whether or not this impacted the iDFS results remains unknown. As of this time, no updates or biomarker analyses from this study have been presented or published.

Subsequently, a number of prospectively designed trials to address this issue have been reported, demonstrating no significant benefit with the addition of an anthracycline to taxane therapy. The West German Study Group phase III PlanB study successfully demonstrated the non-inferiority of a non-anthracycline, taxane-regimen^110^. This prospective, randomized study evaluated whether six cycles of adjuvant TC is non-inferior to four cycles of EC followed by four cycles of docetaxel (100 mg/m2) in patients with HER2− breast cancer (*N* = 2449). Forty percent of patients had LN+ disease, 17% had centrally confirmed TNBC, and 42% had high-grade tumors. With a median follow up of 60 months, the 5-year DFS was 89.6% for EC-T vs 89.9% for TC (TC vs EC-T HR = 0.996) and within the non-inferiority margin. In contrast to the ABC study, subset analysis indicated a similar DFS in each treatment arm regardless of recurrence score, LN status, grade, or TNBC subtype.

Another study, SUCCESS-C, was also undertaken to prospectively evaluate this question. In this 3642-patient phase III trial, treatment with three cycles of FEC followed by three cycles of D was compared to TC for six cycles. A pooled analysis of this trial with the results of PlanB (above) were presented in 2018^111^. Of 5923 patients included, 2979 were assigned to non-anthracyline and 2944 to anthracycline; 52% had LN+ disease, 40% had grade 3 disease, and 22% had TNBC. With 62 months median follow up, DFS for the two arms were almost identical (HR 1.04, *p* = 0.64) and remained similar regardless of luminal subtypes or triple-negative status. The only group that appeared to benefit from anthracycline-based therapy were those with four or more involved LN (DFS 75% for non-anthracycline and 82% for anthracycline).

A smaller (*N* = 650) phase III, a non-inferiority study run by the Hellenic Oncology Research Group (HORG) evaluated 3-year DFS of dose-dense FEC-D (eight cycles total) vs six cycles of TC in women with LN positive disease^112^. Notably, over one-third of patients had at least four LN involved. The 3-year DFS was 89.5% with FEC-D and 91.1% with TC (HR 1.147, *p* = 0.568), though non-inferiority was not met.

Taken as a whole, these studies indicate the benefit of adding an anthracycline to taxane-based chemotherapy in HER2− disease appears to be marginal at best, especially in HR+ disease, and likely is restricted to patients with four or more LN involved.

### Studies comparing taxane/anthracycline vs taxane exclusively in TNBC

Given the poor prognosis associated with TNBC and the lack of targeted systemic therapy options, the choice of a chemotherapy regimen to reduce the risk of distant recurrence may be of particular importance. It is interesting to note that early analyses in the pre-taxane era did not clearly indicate the TNBC subtype uniquely benefits from anthracyclines. For example, a Korean registry analysis^113^ of 4033 patients who had node-negative, triple-negative breast cancer treated with CMF (29.5%), AC (35.2%), FAC (21.7%), or no chemotherapy were evaluated for survival outcomes. While receipt of chemotherapy was significantly associated with improved OS compared to no chemotherapy, there was no difference in survival when comparing the three adjuvant regimens to one another. Similarly, an exploratory analysis of the MA-5 trial evaluating outcomes based on PAM-50 intrinsic subtypes indicated basal-like breast cancer does not benefit from anthracycline and may in fact benefit more from CMF chemotherapy^86^. In contrast, subgroup analysis of the ABC trial in which taxane combinations were utilized^109^ appears to indicate that TNBC derives more benefit from an anthracycline (TC vs TAC TNBC HR 1.42; hormone receptor+ HR 1.12) and is most apparent in TNBC with nodal involvement.

In the past several years, encouraging results have been reported for anthracycline-free, taxane plus platinum chemotherapy regimens for early-stage TNBC. Combined results from two prospective cohorts (University of Kansas and Spain) including 190 patients with TNBC, more than half of whom had LN+ disease, treated with six cycles of neoadjuvant docetaxel and carboplatin (TP) reported^114^ a pCR rate of 55% and Residual Cancer Burden (RCB) 0 + 1 (pCR plus near pCR) rate of 68%. Rates were similar regardless of *BRCA* mutation status. The estimated 3-year RFS were 79% and 3-year OS was 87%^115^. Importantly, the 3-year RFS and OS for those who achieved pCR were 90 and 94%, respectively. Use of adjuvant anthracycline-based chemotherapy was rare in those with pCR (5/100). Nearly 60% of patients with the significant residual disease received anthracyclines postoperatively but this was not associated with a difference in RFS or OS.

A single-arm phase II Peruvian study evaluated pCR rates associated with TP for six cycles (*N* = 27) vs historical controls treated with standard AC for four cycles followed by 12 weekly doses of paclitaxel (*N* = 34) for high-risk TNBC^116^ Over three-quarters had LN involvement and those treated with TP had a significantly larger median tumor size (72.8 vs 52.2 mm, *p* = 0.007). Despite this, the pCR rate was 37% in the TP arm and 23.5% in the AC-T arm and 2-year DFS (73.1 vs 59.3%) and OS (84 vs 71%) were numerically higher with TP.

To date there have been at least six prospective randomized phase II or III clinical trials, three adjuvant and three neoadjuvant (including one led by Sharma and colleagues)^117^, comparing an anthracycline-free, taxane/platinum regimen to a taxane/anthracycline-based regimen in early-stage TNBC (Table 4). All demonstrated either similar or improved outcomes with the non-anthracycline-based regimen^117–122^. Though each of these individual trials was relatively small in size, as a whole they provide data from a total of 828 patients with TNBC, failing to demonstrate that this disease subtype derives a significantly greater benefit from the use of an anthracycline/taxane vs a taxane/platinum-based, non-anthracycline regimen. However, the fact that the TNBC subtype, which accounts for only 10–15% of breast cancer, is comprised of molecularly heterogeneous subtypes makes interpreting efficacy outcomes with cytotoxic chemotherapy in this group of tumors even more challenging.

**Table 4:** Prospective randomized trials of taxane/platinum vs taxane/anthracycline in early triple-negative breast cancer.

Study (Author/Year)	Ref	Treatment	Dz Setting	Phase	*N*	Outcomes
Zhang P 2016 (published)	117	Six cycles of q3w Paclitaxel (175 mg/m^2^ q3) carboplatin (AUC 5) vs Paclitaxel (175 mg/m^2^) Epirubicin (75 mg/m^2^)	NAC	II	91	pCR non-anthracycline: 39% anthracycline: 14% (*p* = 0.014) 3-year RFS (median follow up 55-months) non-anthracycline: 81% anthracycline: 62% (*p* = 0.043)
Najafi S 2017 (published)	118	Q3w Docetaxel (70 mg/m^2^) plus Carboplatin (AUC 7) × 6 vs ACx4→docetaxel/carboplatin (same doses above) × 4	ADJ	II	119	Median follow up 40 months 2-year DFS non-anthracycline: 93% anthracycline: 83% 2-year OS non-anthracycline: 97% anthracycline: 91% Estimated 5-year DFS non-anthracycline: 85% anthracycline: 64%, HR = 2.31; *p* = 0.028 5-year OS non-anthracycline: 92% anthracycline: 81%
Wang J 2019 (abstract)	118	Dose-dense paclitaxel/carboplatin vs Dose-dense EC→paclitaxel	ADJ	III	132	3-year DFS non-anthracycline: 94% anthracycline: 78%, HR = 0.305, *p* = 0.0046 3-year OS non-anthracycline: 98% anthracycline: 93%, *p* = 0.0268
Du F 2020 (published)	120	Six cycles q3w Carboplatin AUC 5 Docetaxel 75 mg/m^2^ or paclitaxel 175 mg/m^2^ vs four cycles EC (epi: 90 mg/m^2^)→ docetaxel (75 mg/m^2^) or paclitaxel (175 mg/m^2^)	ADJ	II	308	Median follow up 66.9 months 5-year DFS non-anthracycline: 84.4% anthracycline: 85.8% *p* = 0.712 5-year OS non-anthracycline: 93.5% anthracycline: 94.4%, *p* = 0.770
Zhang “NeoCART” 2020 (abstract)	121	Six cycles q3w Docetaxel 75 mg/m^2^ Carboplatin AUC 6 vs Epirubicin (90 mg/m^2^)/Cytoxan x 4 → docetaxel (100 mg/m^2^) x 4	NAC	II	88	pCR non-anthracycline : 61% anthracycline : 39%, *p* = 0.033
Sharma P “NeoSTOP” 2021 (published)	116	Six cycles Docetaxel 75 mg/m^2^ q3w Carboplatin AUC 6 q3w vs Paclitaxel 80 mg/m^2^ qw x 12 Carboplatin AUC 6 q3wx4→dose dense ACx4	NAC	II	100	pCR: 54% both arms RCB 0/1: 67% each arm

*A* doxorubicin, *ADJ* adjuvant, *AUC* area under the curve, *C* cyclophosphamide, *DFS* disease-free survival, *E* epirubicin, *HR* hazard ratio, *MBC* metastatic breast cancer, *NAC* neoadjuvant chemotherapy, *pCR* pathologic complete response, *OS* overall survival, *RCB* residual cancer burden index, *RFS* relapse-free survival.

## Selecting patients for anthracyclines: Topoisomerase II alteration

Given the differential data regarding the benefit of anthracyclines in both HER2− and HER2+ breast cancer as well as the clear preclinical data showing that transfection of the *HER2* gene itself does not impart sensitivity to anthracyclines, the search for predictive biomarkers continues. Topoisomerase II alpha (*TOP2A*) codes for a critical enzyme involved in DNA replication^123,124^ and has been identified as a candidate gene for three reasons. First, it is located on the long arm of chromosome 17 in relatively close proximity to the *HER2* locus and sometimes is co-amplified with *HER2*. Second, its protein product (TopoIIα) is a direct target of anthracycline chemotherapy. Finally, *TOP2A* amplification has been shown preclinically to be associated with increased protein expression and increased sensitivity to anthracycline chemotherapy^123,125,126^ Conversely, deletion of *TOP2A* has been associated with decreased expression of TopoIIα and resistance to anthracycline^125,127^.

Before discussing the data relating to topoisomerase amplification/expression with response to anthracyclines, it is important to review evidence relating to the incidence of *TOP2A* amplification in relation to *HER2* amplification, and to highlight how both testing techniques and result interpretation for topoisomerase have varied, leading many to draw conflicting and confusing conclusions.

### TOP2A amplification and relation to HER2 amplification

Interpretation of a number of studies evaluating *HER2* and *TOP2A* amplification have been complicated by the definitions used for amplification. For example, two early studies evaluating *HER2* and *TOP2A* alterations by FISH in breast tumors (*N* = 136^128^ and *N* = 97^125^) indicated that over 40% of *HER2* amplified breast cancers have *TOP2A* co-amplification. This high incidence may be related to the fact that authors defined amplification for both genes as a copy number ratio of >1.5, rather than 2.0^125^. In *HER2* normal tumors, no *TOP2A* alterations were detected. An analysis of samples from the BR9601/NEAT^84^ demonstrated 9 of 26 *TOP2A*-amplified tumors were *HER2*-normal by FISH. However, again the cutoff ratio set for *TOP2A* amplification was >1.5 so tumors that were not actually amplified (defined as >2.0) were likely included. Similarly, two Polish series indicated amplification of *TOP2A* occurs in a substantial proportion of HER2 non-amplified cancer, however again the definition of amplification (TOP2A/CEP17>1.25) undoubtedly led to the counting of *TOP2A* non-amplified tumors as amplified^129,130^.

At least ten other studies evaluating alterations in these two genes demonstrated that *TOP2A* is only amplified in the presence of HER2 amplification^131–140^ and an additional 12 studies reported *TOP2A* amplification in very few cases without HER2 amplification^141–153^. Taken together, the incidence of *HER2/TOP2A* co-amplification from these studies was roughly 35%. The largest of these analyses evaluated 4943 breast cancers, all tested by FISH in one academic central laboratory (USC, Los Angeles, CA, USA) using methods and probes validated by the physical mapping on the 17q12-q21 amplicon^137^. Both *HER2* and *TOP2A* amplification were defined as a copy number ratio to centromere 17 (CEP17) of ≥2.0. The test set consisted of 339 tumors from patients with MBC treated on the registrational trastuzumab (H0648) trial. Of these, 279 were confirmed to be *HER2* positive by FISH, 99 of which were *TOP2A* co-amplified (35%). No *TOP2A* amplification was observed in *HER2* normal tumors. An additional 4604 tumors from the Breast Cancer International Research Group (BCIRG005, *N* = 1614 and BCIRG006, *N* = 2990) trials served as the validation cohort. Again, all tumors were confirmed to be *HER2*-non-amplified (BCIRG005) or *HER2*-amplified (BCIRG006) by FISH in the same central laboratory, using validated probes and methods as well as the FDA approved cutoff for *HER2* amplification. Amplification of *TOP2A* was detected in 35% (1057/2990) of *HER2*-amplified tumors. Not a single case of *TOP2A* amplification was detected in 1614 *HER2* normal tumors.

In addition to differing cutoffs for defining amplification, other factors may account for the fact that some studies have reported *TOP2A* amplification in *HER2* normal tumors including the source of tissue for testing, differing assays (qPCR vs FISH), and lab-to-lab variations leading to discordant results. One example that highlights these issues is a meta-analysis of individual patient data from five studies where *TOP2A* was evaluated. Samples from the Belgian trial, MA-5, DBCG 89D, BR9601, and NEAT trials were included^87^. It is notable that in the original analysis of the Belgian trial^78^, *TOP2A* amplification was defined as a copy number ratio of at least 1.5 and in the BR9601 and NEAT trials^84,85^ it was defined as a copy number ratio of >1.5. In this meta-analysis, however, the definition for *TOP2A* amplification changed to ≥2 without an explanation to support the use of either 1.5 or 2. The investigators originally planned to have all tumors from these five trials retested for *TOP2A* and *HER2* centrally at an external laboratory (University of Tampere, Finland). However, discordance in results from Tampere and the four national laboratories that performed the original analyses for these five trials was noted and thought to be due to the use of tumor sections cut from TMA. When the external laboratory at Tampere used whole tumor sections, the concordance rate improved. In the end, only a handful (123/3,102, 3.9%) of samples were tested for *TOP2A* in Tampere and the final concordance between the central lab and the national laboratory was only 69%.

### Measuring topoisomerase expression

While early evidence suggested amplification of *TOP2A* is associated with overexpression of the protein^125^, differentiating overexpression from normal expression in tumor samples has been challenging and demonstrating a correlation between amplification and expression has not yielded consistent results. It is now well recognized TopoIIα is highly expressed in rapidly dividing, high-grade tumors and can thus be a marker of proliferation rate^154–160^. This is consistent with the critical role played by TopoIIα in cell division. TopoIIα is known to be a tightly regulated gene at both the transcriptional and translational levels whose expression varies dramatically during the cell cycle. The variability in expression throughout the cell cycle likely accounts for the lack of clear correlation between gene copy number and protein level^161,162^. For example, a Canadian group evaluating *HER2* and *TOP2A* by FISH and their protein products by IHC in 81 breast tumor samples observed no correlation between *TOP2A*-amplification and TopoIIα protein expression^145^. Other groups also demonstrated a poor correlation between *TOP2A* gene amplification and TopoIIα protein expression^130,149,153,158,159,163,164^. Irrespective of this inherent molecular variability, a number of investigators evaluated tumors for TopoIIα overexpression, using a variety of definitions, in order to assess for a correlation with outcome in anthracycline-treated patients^77,84,131,133,136,138,139,165–170^. Given the lack of standardization of interpreting IHC results for TopoIIα, the nonexistence of a clear correlation between amplification and expression and evidence to suggest that outcome with anthracyclines is associated with amplification, not expression level^136^, the below section will exclude those studies where topoisomerase was only evaluated by IHC^165–167^ or mRNA^171^, rather than FISH.

### TOP2A amplification and response to anthracyclines

Numerous retrospective analyses (Tables 5 and 6) have been conducted to evaluate whether *TOP2A* amplification is associated with response to anthracyclines. While the majority have indicated either a significant association or a trend between *TOP2A* amplification and benefit from anthracycline, a handful have not. As a whole, the results are difficult to interpret due to their retrospective nature, generally small sample sizes as well as their non-standardized testing techniques and varying definitions of alterations.

**Table 5:** Studies evaluating the relationship between topoisomerase IIa and response to anthracycline (all received anthracycline).

Study	Ref	Regimen	Dz Setting	*N*	Outcomes	Topo2 test/definitions
Jarvinen, et al 1998 Tampere University Finland	Br J Cancer	Epirubicin	MBC	55	TopoIIα expression stable from primary to metastatic sample. Validated IHC Ab test on FFPE. Expression TopIIα (at different levels) does not predict response to first-line epirubicin. FISH not done. HER2 overexpression, lower response to epirubicin.	IHC only rabbit polyclonal Ab; Analyzed as a continuous variable; compared outcome for IHC > or <15%
Rush - Chicago (Retrospective series) Coon 2002	130	Anthracycline	NAC	35	Eight HER2 amplified, six TOP2A amplified-all HER2+ favorable response if co-amplified. Expression of TopoIIα is also associated with a favorable response.	FISH: Ratio >2.5 = amplified for HER2 and TOP2A IHC: Nuclear staining scored 0–4 using JH2.7 Ab
Bergonie Institute MacGrogan 2003	BJC 89 666	EVM-MTC	NAC	125	All received epirubicin based therapy; TopoIIα expression predictor of clinical response	IHC HER2+ if 10% moderate- strong+ IHC KiS7 Ab for Topo2a, high >15%
Royal Marsden series Arriola 2007	138	All anthracycline	Adj	232 of 245	TOP2A amplification only on HER2+ (20/37 HER2+TOP2A+) In HER2+ tumors, TOP2A amplification associated with better OS and DFS Median DFS 115 mos vs 68 mos *p* = 0.026 Median OS 119 mos vs 94 mos, *p* = 0.028 No association with TopoIIa expression and outcome	CISH TOP2A and HER2 Positive if >6 signals/nucleus in >50% cancer cells or when large gene copy clusters were seen IHC with Ki-S1 Ab
HeCOG pooled analysis HE10/97 and HE10/00 Fountzilas 2013	147	E-CMF or E-T-CMF	Adj	1031 of 1681	244 HER2 amplified, 102/244 (42%) co-amplified for TOP2A 102/104 TOP2A amplified were HER2 amplified. 40% had CEP17 gain HER2 amplification, TOP2A amplification and CEP17 gain and HER2/TOP2A co-amplification were not associated with TTR or time to death TOP2A deletions did not result in lower TopoIIa expression. No signif association between TOP2A amplification and expression 5% tumors TOP2A deleted	HER2 >2.2 or copy number >6 TOP2A >2.0 or copy number >6 Deletion <0.8 CEP17 gain if >3 CEP signals in >30% nuclei
Milan case series Orlando 2008	171	E or A	NAC	23 (of 286)	TOP2A amplification predicts pCR after neoadjuvant anthracycline-based therapy (epirubicin or doxorubicin) in ER/PR negative, HER2 overexpressing. 22% (5/23) TOP2A amplified. 4/23 del. 1 chromosome 17 polysomy. pCR 60% (3/5) in TOP2A amp; 17% (3/18) in TOP2A non-amplified. 0/4 TOP2A deleted tumors pCR; Disease progression in one TOP2A deleted tumor.	FISH Ratio >2 = positive Ratio <0.8 = del
CALGB 8541 Harris 2009	143	CAF	ADJ	624	HER2+ 117/624 = 19%. TOP2A amp 41 (7%), del 69 (11%); 39/41 HER2/TOP2 coamplified TOP2A amp does not predict benefit from CAF in HER2+. 41 patients TOP2A+ spread over three arms; All pts received anthracycline. No info regarding the distribution of patients across dose strata (very small numbers in each of three dose strata, underpowered).	FISH HER2 & TOP2A Ratio >2 = positive Ratio <0.67 = del
SWOG S9313/INT0137 Tubbs 2009	142	AC vs A→C	ADJ	1729 Of 3125	1380 patients had both HER2 and TOP2 FISH done. 279/1483 (18.8%) patients HER2 amplified; 65/1626 TOP2A+; 64/65 TOP2+/HER2+. No association between TOP2 alteration and outcome to anthracycline therapy. Positive association with HER2.	FISH HER2 and TOP2ARatio >2 = positive Ratio <0.7 = del
BCIRG005 Press 2011	136	AC-T TAC	ADJ	1614	HER2 neg by FISH. 2.6% TOP2A deleted (42/1614). Not one had TOP2 amplification	FISH HER2 and TOP2A Ratio >2 = positive <0.82 = deleted
TOP Trial Desmedt 2011	135	Epirubicin all patients	NAC	149	ER negative treated with epirubicin monotherapy to look at predictive value of TOP2A. 10/106 samples TOP2A amplified, all HER2 amplified. 33/106 HER2 amplified: higher chance of pCR if HER2 amplified. TOP2A amplification (not expression) associated with pCR (*p* < 0.001). Developed anthracycline-based score (A-score) to combine Top2A gene signature with tumor invasiveness and immune response signatures	FISH Ratio >2 = positive Ratio <0.8 = del
Chen 2013	Eur J Surg Oncol 39:619-26	Anthracycline	NAC	99	Topo2a expression associated with pCR with anthracycline-based neoadjuvant therapy	IHC 10% = positive

*A* doxorubicin, *Ab* antibody, *ADJ* adjuvant, *amp* amplification, *BCIRG* Breast Cancer International Research Group, *CALGB* Cancer and Leukemia Group B, *C* cyclophosphamide, *CEP17* chromosome enumeration probe 17, *CISH* chromogenic in situ hybridization, *del* deleted, *DFS* disease free survival, *E* epirubicin, *ER* estrogen receptor, *F* fluorouracil, *FISH* fluorescent in situ hybridization, *FFPE* fresh frozen paraffin embedded, *HD* high dose, *HeCOG* Hellenic Cooperative Oncology Group, *HR* hazard ratio, *IHC* immunohistochemistry, *M* methotrexate, *MBC* metastatic breast cancer, *NAC* neoadjuvant chemotherapy, *NR* not reported, *OS* overall survival, *P* melphalan, *pac* paclitaxel, *pCR* pathologic complete response, *p-int* p-value interaction test, *PR* progesterone receptor, *RFS* relapse-free survival, *SWOG* Southwest Oncology Group, *T* docetaxel, *V* vincristine.

**Table 6:** Studies evaluating relationship between topoisomerase IIa and response to anthracycline vs non-anthracycline or differing doses of anthracyclines.

Study	Publication	Regimens	Setting	*N*	Outcomes	Topoisomerase 2a test/definitions
Belgian Di Leo 2001	77	CMF vs EC vs High dose EC	ADJ	481	Most samples had 1–25% of cells positive for TopoIIα. 161 tumors TopoIIα positive; only 25 were HER2 positive by IHC TopoIIα expressing trended toward improvement in EFS with an anthracycline-based adjuvant therapy. 50 TopoIIα + High-EC vs CMF: HR = 0.66, *p* = 0.25; 50 TopoIIα- High-EC vs CMF: HR = 1.26, *p* = 0.51; *p* interaction 0.13	IHC only KiS1 Ab >10% is positive HER2 FISH >2 = positive
Belgian Di Leo 2002	78	CMF vs EC vs High dose EC	ADJ	61	23/61 of HER2+ were TOP2A+ (did not look for TOP2A in HER2 negative) Trend toward EFS benefit with EC or High dose EC only in HER2+ tumors Co-amplified had better EFS with EC or High dose EC vs CMF; TOP2A negative did not appear to benefit from anthracyclines	FISH HER2+ ratio >2.0 TOP2A+ ratio >1.5 TOP2A ratio <0.8 del
Belgian Case-Control Cardoso 2004	132	Anthracycline vs taxane	MBC	58	TOP2A amplification in eight tumors. All were HER2+ TOP2A+ treated with anthracycline: 21% CR; 6% progressive disease; TOP2A+ treated with taxane: 14% CR and 14% progressive disease	FISH HER2 ratio >2.0 TOP2A ratio >1.5 positive IHC >10%
DBCG 89D* Knoop 2005	81	CEF vs CMF	ADJ	805	246 HER2 positive by IHC or FISH; 79 of these (32%) TOP2A co-amplified. 14/527 HER2 negative (IHC), TOP2A amplified Anthracycline benefit similar in HER2+ and HER2−. TOP2A amplification had improved RFS (HR = 0.43) and OS (HR = 0.57) with CEF. TOP2A del may also associate with outcome.	FISH HER2 and TOP2A ratio >2 positive TOP2A ratio <0.8 del
DBCG 89D* Nielsen 2008	146	CEF vs CMF	ADJ	767	Updated analysis of TOP2A aberrations with IVD-labeling; TOP2A status/*N*: Amplified: *N* = 92 (12%); Deletion 87 (11%) Normal: *N* = 589 TOP2A normal have longer RFS than TOP2A amp or del RFS CEF vs CMF: TOP2A amplified: HR = 0.39, *p* = 0.002; TOP2A deleted: HR = 0.61, *p* = 0.083; TOP2 negative: HR = 0.94, *p* = 0.60 OS CEF vs CMF: TOP2A amplified: HR = 0.48, *p* = 0.01; TOP2A deleted: HR = 0.68, *p* = 0.155; TOP2 negative CEF vs CMF: HR = 0.90, *p* = 0.42	FISH HER2 and TOP2A ratio >2 positive TOP2A ratio <0.8 del IVD-labeling of TOP2A FISH pharmDx™ Kit
DBCG 89D* Ejlertsen 2010	J Clin Oncol 2010;28:984-90	CEF vs CMF	ADJ	623	Looked at TIMP1, HER2 and TOP2A and association with anthracycline benefit. Combining TOP2A abnormal with TIMP1 negative expression (2T responsive profile) predicted better iDFS and OS with CEF vs CMF. Stronger predictor than HER2+ ± TIMP1−. underpowered	FISH HER2 and TOP2A ratio >2 positive TOP2A ratio <0.8 del
Scandinavian Breast Group 9401 Tanner 2006	174	FEC × 9 (Tailored/dose escalated) vs FEC × 4 followed by ASCT (less anthracycline)	ADJ	391 of 525	HER2 by CISH, TOP2A checked in HER2+ (*N* = 128) only Significantly shorter RFS and OS in HER2+; 37.5% (48/128) TOP2A coamplified. Patients with TOP2A-amplified tumors had a statistically significantly better breast cancer RFS when treated with tailored and dose escalated FEC (high dose anthracycline) compared with standard FEC-ASCT (HR = 0.45; *P* = 0.049); no such difference between FEC9 and ASCT in patients with no TOP2A amplification (HR = 0.95; *P* = 0.88). Multivariate cox regression analysis showed among TOP2A amplified, FECx9 treatment was the only significant predictor for a better outcome (adjusted HR = 0.30; P = 0.020). For tumors without TOP2A amplification, treatment did not predict outcome in multivariate analysis.	CISH Ratio > 2 or >6 copies in >30% of nuclei or presence of an easily identifiable gene copy cluster, if gene copies could not be counted
TAX303 Durbecq 2004	164	A vs T	MBC	108	MBC: 43 patients Topo2 protein positive, higher response to anthracyclines if Topo2+. No diff in TTP and OS Both 2004 and 2007 analyses show similar results by expression	IHC: TopoII α-positive: >10% of immunostained cells
TAX303 Di Leo 2007	165	A vs T	MBC	91	36/91 TOP2A+; The probability of response to doxorubicin is higher in topoII α-positive than in topoII α-negative tumors [Odds ratio 4.51 (95% CI 1.01–20.10), *P* = 0.05]. This correlation is not found for docetaxel [OR 0.75 (95% CI 0.21–2.75), *P* = 0.66]. Response to anthracycline is highest (5x better) for p53 wild type/Topo2a+ (*N* = 16) vs other pts	See above
MA-5 O’Malley 2009	149	CEF vs CMF	ADJ	438 (62%)	33/116 HER2+ TOP2A+ (28%); 20/314 HER2 neg, TOP2A amp 12% (54/438); TOP2A deletion 6% (26/438) TOP2A amplification trended toward a relationship with better RFS and OS with CEF than CMF but not statistically signif. 5-year RFS CEF vs CMF TOP2A+ (*N* = 54): HR = 0.51, *p* = 0.20; TOP2A del (*N* = 26): HR = 0.16, *p* = 0.02; TOP2A normal (*N* = 358): HR = 0.90, *p* = 0.49; *p* interaction 0.22 5-year OS CEF vs CMF TOP2A+: HR = 0.47, *p* = 0.20; TOP2A del: HR = 0.18, *p* = 0.07; TOP2A normal: HR = 1.09, *p* = 0.62; *P* interaction 0.07 After adjustment, HR interaction between treatment and altered vs normal TOP2A 0.53 for RFS (*P* = 0.09) and 0.38 for OS (*P* = 0.02).	FISH HER2 and TOP2A ratio >2 positive TOP2A ratio <0.8 del
BR9601 Bartlett 2008	84	CMF vs E-CMF	ADJ	303	No association between TOP2A amplification or alteration in RFS and OS; No association between TOP2A amp/alteration and outcome with anthracycline. Definition of TOP2A amplification different from HER2. 63 HER2 amplified; worse RFS and OS with epirubicin 26/303 TOP2A amplified (9%), 17/26 coamplified for HER2; nine TOP2A+, HER2 Nl (may be due to TOP2A amplification definition) TOP2A deletion 50/303 (17%) – did not evaluate outcome or interaction based on this alone	FISH: HER2 ratio >2.0; TOP2A ratio >1.5 positive TOP2A ratio <0.8 del IHC: >median
BR9601/NEAT Bartlett 2010	85	CMF vs E-CMF	ADJ	1762	367 HER2 amplified (21%); 169/1762 TOP2A amplified (10%); 191/1762 TOP2A deleted (11%). Unknown how many are co-amplified. -HER2 amp or TOP2A del worse RFS/OS. TOP2A amp and CEP17 dup no relation to RFS/OS. -no significant interaction with anthracycline benefit for HER2 or TOP2A (del or amplification or combined). -CEP17 duplication predicted RFS and OS benefit from anthracycline (significant interaction) HER2 amp and TOP2A alterations (amp and del) significantly associated with CEP17 dup (*P* < 0.0001)	HER2 ratio >2.0 (different from above) TOP2A ratio >1.5 TOP2A ratio <0.8 del CEP17 dup = >1.86 signals/cell
CALGB 8541 Harris 2009	143	CAF Different doses	ADJ	624 of 1572	HER2+ 117/624 = 19% TOP2A amp 41 (7%), del 69 (11%); 39/41 HER2/TOP2 co-amplified TOP2A amp does not predict benefit from CAF in HER2+. 41 patients TOP2A+ spread over three arms; All pts received anthracycline. No info regarding the distribution of patients across dose strata (very small numbers in each of three dose strata, underpowered).	FISH HER2 & TOP2A Ratio >2 = positive Ratio <0.67 = del
H0648 Press 2011	136	Anthracycline or taxane ± trastuzumab	MBC	339 of 469	279 confirmed to be HER2 amplified; 99/279 coamplified TOP2A (35% of HER2+); 45/279 (16%) del TOP2A in HER2+ No TOP2A amplification in HER2 negative. 2/60 HER2 neg, TOP2A deleted 2/60 HER2 negative. TOP2A/HER2 coamplified: longer PFS/OS compared with normal TOP2A if treated with anthracycline. In non-trastuzumab treated patients: OS better with anthracycline if TOP2A amplified (38.5 mos) vs non-amp (18.2 mos, *p* = 0.004) and OS same with paclitaxel for TOP2A amp (18.4 mos) vs non-amp (20.6 mos) (*p* = NS)	FISH HER2 and TOP2A Ratio >2 = positive; Ratio <0.82 = del Used methods and probes validated from physical mapping of 17q12-q21 amplicon.
BCIRG006 Press 2011	136	AC-T vs AC-TH or TCarboH	ADJ	2990	1057/2990 (35%) HER2/TOP2A coamplified; 145/2990 HER2+/TOP2 deleted If TOP2A amplified, AC-T DFS/OS improved similar to AC-TH and TCH. If TOP2A non-amplified, AC-T does worse, AC-TH = TCarboH	FISH HER2 &TOP2A Ratio >2 = positive; Ratio <0.82 = del
Meta-analysis (Belgian, MA-5, DBCG89b, NEAT, BR9601) Di Leo 2011	87	CMF vs anthracycline	ADJ	3102	3102 tested for TOP2A, 3452 for HER2. 316/3102 (10%) deleted for TOP2A, 275 (9%) TOP2A amplified. Concordance of 123 samples tested at external lab (Tampere) compared to results from university labs: 69% Did not provide % of pts with HER2/TOP2A co-amplification or TOP2A amp in HER2 negative EFS anthracycline vs CMF: TOP2A normal: 0.88 (0.78, 1.00); TOP2A del: 0.63 (0.46,0.87); TOP2A amp: 0.62 (0.43,0.90); *p* interaction = 0.0513 OS anthracycline vs CMF: TOP2A normal: 0.89 (0.78, 1.03); TOP2A del: 0.68 (0.49,0.95); TOP2A amp: 0.67 (0.46,0.98) *p* interaction = 0.1608 when combined deleted/amplified it is statistically significant	FISH Ratio >2 = positive Ratio <0.8 = del
Meta-analysis (see above)	134	CMF vs anthracycline	ADJ	3846	HER2: Total 3436, 832+ (24%); TOP2A: Total 3098, 273+ (9%), 314 Del (10%) CEP17: Total 3225, 971 duplicated (30%) TOP2A del and TOP2A del/amp poorer RFS and OS; Noted heterogeneity for the prognostic impact of some markers across studies Interaction for treatment with HER2 not significant for RFS/OS in adjusted analyses. Stratified by trial, adjusted for confounders, Cox regression model shows interaction for CEP17 dup and anthracycline significant for RFS (0.01), borderline for OS (*p* = 0.06). TOP2A interaction significant for both RFS (*p* = 0.03) and OS (*p* = 0.03)	FISH: Ratio >2 = TOP2A and HER2 positive Ratio <0.8 = TOP2A del Definition CEP17 dup: >1.86 copies per cell for DBCG, BR9601, NEAT, Belgian; >2.25 copies/cell for MA-5
TRYPHAENA Schneeweiss 2014	Br Ca Res 16:R73	FEC/THPer vs FECHPer-THPer vs TCarboHPer	NAC	192	TOP2A amplification (62/192, 32%) not associated with different pCR for FECHPer/THPer; TCarboHPer or FEC-THPer.	FISH: Ratio >2.0 = positive

*A* doxorubicin, *Ab* antibody, *ADJ* adjuvant, amp amplification, *BCIRG* Breast Cancer International Research Group, *CALGB* Cancer and Leukemia Group B, *C* cyclophosphamide, *Carbo* carboplatin, *CEP17* chromosome enumeration probe 17, *CISH* chromogenic in situ hybridization, *CR* complete response, *DBCG* Danish Breast Cancer Cooperative Group, *del* deleted, *DFS* disease-free survival, *dup* duplicated, *E* epirubicin, *ER* estrogen receptor, *EFS* event-free survival, *F* fluorouracil, *FISH* fluorescent in situ hybridization, *FFPE* fresh frozen paraffin embedded, *HD* high dose, *HR* hazard ratio, *IHC* immunohistochemistry, *M* methotrexate, *MBC* metastatic breast cancer, *NAC* neoadjuvant chemotherapy, *NR* not reported, *PFS* progression-free survival, *OS* overall survival, *pCR* pathologic complete response, *Per* pertuzumab, *p int* p value interaction test, *RFS* relapse-free survival, *T* docetaxel, *TTP* time to progression.

Six studies in the neoadjuvant^131,136,152,172–174^ setting have suggested *TOP2A* amplification is associated with better pathologic response to anthracycline-based therapy. Analysis of samples from the adjuvant Scandinavian Breast Group 9401 trial^175^ suggested that patients with *TOP2A* amplified tumors (*N* = 48) had a better RFS when treated with dose escalated FEC compared with standard FEC followed by high dose chemo and stem cell rescue (HR = 0.45, *p* = 0.049). There was no difference in outcome between the two treatment arms in the *TOP2A* normal group. In contrast, analysis of the CALGB 8541 trial^144^, in which patients were assigned to low, moderate, or high (now standard) dose anthracycline, failed to demonstrate an association with *TOP2A-*amplification and benefit from high dose CAF. Only 41 patients spread across three treatment arms had *TOP2A-*amplified tumors and no information was provided regarding how many patients were assigned to each dose level. Therefore, this analysis was conspicuously underpowered. Other retrospective analyses of adjuvant studies in which all patients received the same dose of anthracycline have also been conducted with varying results^139,143,148^.

Press and colleagues analyzed *TOP2A* status on 279 *HER2* amplified tumors from the H0648 trial in which patients were randomized to receive chemotherapy (either AC or paclitaxel) alone or with trastuzumab^137^. They demonstrated a significant association between

*TOP2A* co-amplification and improved survival (*p* = 0.004) in patients treated with an anthracycline. No difference in survival was noted in patients with *TOP2A* amplified or non-amplified tumors who were treated with paclitaxel.

Five larger randomized studies comparing anthracycline vs non-anthracycline chemotherapy were also analyzed retrospectively for *TOP2A*. Di Leo and colleagues analyzed samples from the Belgian study^77,78^ comparing adjuvant HEC to either standard dose EC or to CMF. Benefit with HEC vs CMF appeared to be restricted to those with TopoIIα expression (HR 0.66) compared to those without expression (HR 1.26), (*p* interaction = 0.13). Similar trends were reported when comparing HEC vs EC. The investigators went onto evaluate 61 HER2+ tumors for *TOP2A* amplification by FISH, defining amplification as a gene copy:CEP17 ratio of >1.5. For those with *HER2/TOP2A* co-amplification, EFS was better with anthracycline-containing arms (HEC or EC) vs CMF. Those without *TOP2A* amplification did not appear to benefit from anthracycline.

Samples from the MA-5 study were also evaluated for *TOP2A*^150^. While *HER2* had been determined in 639 (90%) of tumors from whole sections, *TOP2A* was able to be determined for only 438 (62%) of samples. A clear trend toward differential RFS and OS benefit with anthracycline-based therapy in *TOP2A* amplified (ratio >2) cancers was noted but did not meet statistical significance. The hazard ratio for RFS for CEF vs CMF in those with *TOP2A* amplified tumors (*N* = 54) was 0.51 (*p* = 0.20) and was 0.90 for *TOP2A* normal (*N* = 358) tumors.

A larger analysis was performed on samples from the DBCG 89D trial in which patients were treated with adjuvant CMF or CEF^81,147^. While there was no differential benefit from anthracycline-based therapy in patients with *HER2*+ vs *HER2*− breast cancer in this study, those with tumor *TOP2A*-amplification (*N* = 92) (ratio >2) had a significantly improved RFS and OS with CEF. Those with *TOP2A*-normal tumors (*N* = 589) derived no differential benefit with anthracycline-based therapy.

Two combined analyses of samples from the BR9601 and NEAT studies, in which patients were treated with adjuvant CMF or CMF-epirubicin were also published^84,85^. There was no association between *TOP2A*-amplification and differential benefit from anthracycline. However, as previously pointed out, the cutoff for *TOP2A* amplification was set at a gene copy ratio of >1.5. rather than the more accepted >2. As a result, a number of *TOP2A* normal tumors were likely included in the *TOP2A* “amplified” group.

Two meta-analyses^87,176^ were conducted on the same five similarly designed studies comparing adjuvant CMF vs anthracycline-based chemotherapy (DBCG 89d, BR9601, NEAT, MA-5, and the Belgian trial) to address whether the *TOP2A* alteration predicts benefit from an anthracycline. The first^87^, published in 2011, is described in detail above and demonstrated that the improved outcome associated with anthracyclines appeared to be restricted to patients with *HER*2-amplified tumors. Of 3102 samples tested for *TOP2A*, 275 (9%) were amplified (ratio >2). Those with *TOP2A-*amplification showed a greater benefit from anthracycline vs CMF chemotherapy (EFS HR = 0.62, OS HR = 0.67). In contrast, no differential benefit was observed for anthracycline-based therapy vs CMF in the 2511 patients with *TOP2A-*normal tumors (EFS HR = 0.88, OS HR = 0.89). As pointed out above, testing a portion of these samples at a central laboratory showed a relatively low (69%) concordance in *TOP2A* results, calling into question the reliability of these results. The authors acknowledge this by calling for increased standardization of *TOP2A* FISH testing and advising against the routine use of *TOP2A* testing to select patients for anthracycline-based treatment.

### TOP2A deletion

From a biological standpoint, it does not make intuitive sense that patients with tumor *TOP2A* deletion would be more sensitive to anthracyclines. Indeed, early studies indicated deletion of *TOP2A* is associated with diminished expression of the protein and resistance to anthracycline^125,127^. However other studies have indicated that deletion of *TOP2A* does not correlate with reduced expression of TopoIIα^148,177^. Once again, the close connection between proliferation and TopoIIα expression during cell division only complicates the matter. In addition, analysis of tumor samples from several trials^81,87,147,150^ have reported that deletion of *TOP2A* may be associated with increased sensitivity and better outcome with anthracycline vs non-anthracycline-based therapy, though this finding is not consistent^85^. In the DBCG 89d, *TOP2A* deletions were reported in ~11% and trended toward benefit from anthracycline-based therapy. When combining patients with amplification and deletion (“*TOP2A* altered”), this interaction between treatment and marker became significant^81,147^. An analysis of these samples using PAM-50 intrinsic subtyping published in 2020^88^ indicated that two-thirds of tumors designated as *TOP2A* deleted were actually HER2-enriched. Samples from the MA-5 trial^150^ were also tested and only 26 of 438 (6%) were determined to be *TOP2A* deleted. Those with deletion appeared to gain substantial benefit from anthracyclines (RFS HR = 0.16 *p* = 0.02, OS HR = 0.18, *p* = 0.07), though the small sample size should be noted. When patients with either *TOP2A* alteration were combined, a significant benefit with CEF over CMF was observed (adjusted RFS HR = 0.35, *p* = 0.005; adjusted OS HR = 0.33, *p* = 0.008) whereas those with *TOP2A* normal tumors did similarly whether treated with CMF or CEF. The test for interaction between treatment and *TOP2A* status (altered vs not) trended toward significant RFS (*P* = 0.09) and was significant for OS (*P* = 0.02). Both these studies defined *TOP2A* deletion as a ratio of <0.8. In contrast, the BR9601/NEAT analysis^85^ in which 11% (191/1762) patients had TOP2A deletion (ratio <0.8), demonstrated no significant interaction with benefit from anthracycline treatment and *TOP2A* del, *TOP2A* amplification, or *TOP2A* alteration. A meta-analysis including the above four studies plus the Belgian trial also looked at *TOP2A* deletions^87^. Of 3102 tumors, 316 (10%) were *TOP2A* deleted (*TOP2A/CEP17* ratio <0.8). Patients with *TOP2A* deletions seemed to benefit greater from anthracycline compared to those with normal tumors. Again, when all patients with *TOP2A* alterations were combined and compared to those with *HER2* normal tumors, there was a significant interaction in favor of anthracyclines for *TOP2A* altered tumors (EFS *p* interaction = 0.0183, OS *p* interaction = 0.0455). However, the lack of biological rationale to explain how *TOP2A* gene deletion might be associated with anthracycline benefit makes one wonder if there is a different explanation for this effect observed on retrospective studies, each with small patient numbers.

### The exploration of other genomic alterations

As referenced above, Desmedt and colleagues analyzed tumor tissue from a neoadjuvant clinical trial of anthracycline-based therapy and showed that pCR was associated with *TOP2A* amplification^136^. As part of this study, they also developed an “A-score” comprised of a *TOP2A* gene signature and two signatures related to tumor invasion and immune response. They validated the A-score in two cohorts of patients treated with neoadjuvant anthracycline-based regimens. This signature was shown to be associated with a high negative predictive value for pCR in both HER+ and negative disease. It does not appear, however, to have been evaluated in a study with anthracycline- vs non-anthracycline-based therapy.

Investigators have also investigated duplication of chromosome 17 centromere (CEP17 dup) as a marker of sensitivity to anthracyclines. It should be noted that it is not rare to see increased numbers of CEP17 in breast cancer and, in particular, HER2+ breast cancer (Michael Press, MD, PhD, personal communication). It is not clear that these increased CEP17 numbers actually represent duplication. It is difficult to envision why increased copies of CEP17 would lead to anthracycline sensitivity. However, a number of groups analyzed breast tumor samples for CEP17 dup with varying definitions (e.g., >1.86 CEP17/cell or >2.25 CEP17/cell) with inconsistent results^176,178,179^.

It should be acknowledged that our understanding of the evolution of genomic alterations in cancer continues to advance. For example, whole genome sequencing, with its enriched view of genomic structure, has uncovered mechanisms, such as chromothripsis-or the rapid accumulation of hundreds of gene rearrangements over a very short period of time, that lead to silencing of some genes and the generation of “neo”chromosomes that may become focally replicated oncogenic drivers by a process now termed, “chromanasynthesis”^180–183^. These events may lead to a high degree of genomic complexity and drive the evolution of tumor response or resistance to therapy. Whether and how these types of alterations may impact the sensitivity of breast cancer to different types of chemotherapy has not yet been elucidated, though work in this area will hopefully shed further light.

## First prospective evaluation of anthracycline vs non-anthracycline in *TOP2A* normal disease

All the above analyses relating to topoisomerase were performed retrospectively on tumor tissue from patients primarily treated with non-taxane-based chemotherapy. Tissue source, tumor quality, differing definitions of gene amplification, and interlaboratory discordance may all have significantly impacted results. Taken together, however, the majority of the evidence seemed seems to indicate that *TOP2A* alterations were associated with anthracycline benefit, but what was needed was a prospectively conducted randomized trial to evaluate whether anthracyclines benefit patients with *TOP2A* normal disease. The DBCG-07 READ trial was just this type of study^184^. Six cycles of docetaxel (75 mg/m^2^) and cyclophosphamide were compared to three cycles of EC followed by three cycles of docetaxel (100 mg/m^2^) in patients with early-stage, high risk, *TOP2A* normal breast cancer. In contrast to previous analyses^87,176^ *TOP2A* status was determined by an FDA approved FISH assay (TOP2A pharmDX; Dako A/S, Glostrup, Denmark) at one of three laboratories using a signal-to-centromere 17 ratio of 0.8–1.9 as the definition of normal. A total of 2012 patients were recruited to the trial and were followed for a median of 69 months. In this prospective study, there was no significant difference between the two groups with regard to DFS (HR = 1.00, *p* = 1.00) distant DFS (HR = 1.12, *p* = .40) or OS (HR = 1.15, *p* = 0.41). No difference in outcome was noted based on estrogen receptor status or Ki67, both of which. were tested locally. Counterintuitively, subset analysis seemed to indicate those with lower-grade tumors and postmenopausal patients benefit more from EC-D. It is unclear if this finding is real and if so, whether this is due to a higher dose of docetaxel or the addition of the anthracycline. Regardless, these data do not support the claim that anthracyclines preferentially benefit those with TNBC or high-grade tumors.

One interesting point to note is that the Danish investigators carried out central *TOP2A* analysis in 5153 patients prior to enrolling to this study and identified 835 with a *TOP2A* alteration. Ongoing analyses are planned to evaluate what percentage of patients had *TOP2A* amplification vs deletion and how the alteration correlated with *HER2* status (personal communication Bent Ejlertsen).

The BCIRG005 analysis of 1614 samples tested centrally for *HER2* and *TOP2A* demonstrated zero cases of *TOP2A* amplification in *HER2* normal tumors^137^. This calls into question whether false positives account for the reporting of *TOP2A* positive, *HER2* normal tumors. Only 42 (2.6%) of tumors were determined to be *TOP2A* deleted in the BCIRG analysis and it was not associated with differential DFS or OS in this study in which all patients received anthracyclines.

## There is no role of anthracyclines in HER2+ disease regardless of *TOP2A* amplification

Though *HER2* amplified tumors displayed varying sensitivity to anthracycline-based regimens described above, the introduction of trastuzumab revolutionized systemic therapy in this patient population. After improved TTP and OS was shown with the addition of trastuzumab to chemotherapy in metastatic HER2 amplified breast cancer^185^, further studies in early-stage *HER2* amplified breast cancer^186–188^ demonstrated significant DFS and OS benefit with the addition of trastuzumab to standard chemotherapy. However, as all three large studies used a combination of trastuzumab and an anthracycline, there was also a four to fivefold increase in the rate of CHF.

A fourth study (BCIRG006) assigned patients with early-stage *HER2* amplified breast cancer to receive either AC-T, AC-T+trastuzumab, or a new anthracycline-sparing, platinum-containing regimen: TCH (docetaxel, carboplatin, and trastuzumab)^189^. This regimen was based on preclinical studies showing synergy with platinum salts and trastuzumab, which was not evident with anthracyclines or taxanes^190–192^. As expected, each trastuzumab-containing group had improved DFS and OS compared to the AC-T arm. Though the statistical plan was to compare each trastuzumab arm head-to-head with the control arm, a post hoc statistical comparison between the two trastuzumab arms was performed, revealing no difference in efficacy with respect to DFS or OS.

Anthracycline-free regimens have also been evaluated in the neoadjuvant setting for HER2+ breast cancer. In 2020, results from neoCART, a phase II neoadjuvant study comparing TCH to EC-TH were presented^122^. Of 131 treated patients, two-thirds had node involvement. The tpCR rate was 56% with TCH and 38.5% with EC-TH (*p* = 0.044). Furthermore, two studies (TRYPHAENA and TRAIN-2) examined neoadjuvant trastuzumab and pertuzumab-containing regimens for HER2 amplified, early-stage breast cancer and showed no significant difference in the rates of pCR or EFS with or without an anthracycline^193–195^. It is notable that in TRAIN-2, patients in the non-anthracycline arm received nine cycles of weekly paclitaxel plus carboplatin with trastuzumab plus pertuzumab. In the anthracycline arm, a total of five chemotherapy agents plus pertuzumab/trastuzumab (5′fluorouracil, epirubicin, cyclophosphamide, carboplatin and paclitaxel, FEC-HP→TCHP) were given. In spite of the fact that the anthracycline arm received more chemotherapy agents, the pCR rate and EFS rates were identical. Some argue that anthracycline-based therapy should be reserved for those with the highest risk of relapse. In contrast, however, subset analyses of BCIRG006 and TRAIN-2 demonstrate that the recurrences for patients with four or more LN involved was not improved by the addition of an anthracycline to trastuzumab-based therapy^195,196^. In fact, the TRAIN-2 study showed a trend toward a better outcome with the non-anthracycline treatment in this high-risk group.

In terms of safety, treatment-related leukemic events were noted in the anthracycline arms of both TRAIN-2 and BCIRG006. Moreover, significantly higher rates of cardiac toxicity were observed in the anthracycline arms of these studies, with sustained cardiac dysfunction noted during follow up.

It is noteworthy that in BCIRG006, all patients had centrally confirmed HER2 amplified tumors. Central testing for *TOP2A* was also performed on samples from 2990 patients and demonstrated that 35% (1057) had *TOP2A* co-amplification and 145 (5%) had *TOP2A* deletion^137^. For *TOP2A* normal tumors, TCH and AC-TH were similarly associated with a significantly improved DFS and OS compared to AC-T. Those with *TOP2A* amplification benefited equally from AC-T, AC-TH, and TCH, suggesting that in *HER2*-amplified tumors with *TOP2A* co-amplification, a similar outcome is achieved with targeting one vs both alterations. Thus, inhibiting both TOP2A (with doxorubicin) and HER2 (with trastuzumab) in the AC-TH arm does not appear to improve efficacy. Some would argue these patients could thus avoid trastuzumab and just use AC-T, while others would conclude that the safer TCH regimen should be used to maximize the therapeutic index.

These trials provide the best evidence that, with HER2-directed therapy, there is no significant benefit of adding anthracyclines to neo/adjuvant regimens in early-stage breast cancer. Accordingly, in 2021, the National Comprehensive Cancer Network Guidelines^197^ removed anthracycline-based therapy from the list of “preferred regimens” for the treatment of HER2+ early-stage breast cancer and into the category of regimens for use in certain situations. One notable situation where an anthracycline would be appropriate to consider is for a pregnant woman diagnosed with a HER2+ breast cancer. In this situation, the use of trastuzumab is contraindicated and the standard approach would be to proceed with anthracycline-based chemotherapy.

## Why do we care? Toxicity of Anthracyclines

One of the most recognized consequences of anthracycline use is myocardial injury. Although heart failure is widely acknowledged to be associated with these drugs, measuring the true incidence of heart damage remains elusive, due to limited long-term studies in asymptomatic patients, the presence of confounders including cardiac risk factors and concomitant use of other cardiotoxic cancer therapies and the retrospective nature of many studies aimed at gauging rates of cardiac dysfunction. The large adjuvant trastuzumab studies provide unique insights into cardiac outcomes in those treated with anthracyclines or anthracyclines followed by trastuzumab. For example, the NCCTG N9831 and NSABP B31 trials reported that 0.6–1.3% of patients treated with AC-T and 3.0–4.0% of patients treated with AC-TH developed a cardiac event during the 6- to 7-year follow up, respectively. Though these rates seem somewhat low, it is important to call out that 5–7% of patients developed a cardiac event during four cycles of AC chemotherapy that precluded them from proceeding to trastuzumab (or placebo)-based therapy. These studies, in which serial left ventricular ejection fraction (LVEF) measurement was required prior to and after treatment with four cycles of AC revealed higher rates of heart damage than was perhaps previously recognized since patients needed to meet certain heart function criteria, even if they had no symptoms of heart damage, in order to proceed onto trastuzumab-based therapy^198,199^. That said, these studies only measured LVEF during or shortly after active treatment (i.e., 18–21 months), thus long-term subclinical cardiac damage is likely underreported. In contrast, BCIRG006, followed all patients with cardiac function measurements long-term. At 10 years, 1.96% of patients assigned to AC-TH, 0.76% of those assigned to AC-T, and 0.37% of those assigned to TCH developed CHF^196^. Importantly, LVEF decline >10% from baseline was noted in 19, 12, and 9% of those treated with AC-TH, AC-T, and TCH, respectively. While the mean drop in LVEF was transient in TCH-treated patients, it did not recover to baseline in those treated with an anthracycline. Keeping in mind clinical trial patients tended to be younger with a low incidence of cardiac risk factors due to screening requirements, it is likely these cardiac outcomes would be worse in a real-world setting.

In addition to the more common cardiac effects of anthracyclines, another life-threatening complication is therapy related myelodysplastic syndrome and acute myeloid leukemia (t-MDS/AML) resulting from acquired somatic mutations in hematopoietic stem/progenitor cells. Topoisomerase II inhibitors contribute to leukemogenesis by inducing chromosomal breakages and translocations, in regions known to cause malignant transformation, such as 11q23, 21q22, inv(16), t(15,17), and t(9,22)^200,201^. Large studies suggest that the overall incidence of anthracycline-associated t-MDS/AML, while greater than that of the general population, remains <1%, with 10-year cumulative risks ranging from 0.2–1.7%^202^. This risk seems to increase with a greater cumulative dose of anthracyclines, especially when combined with cyclophosphamide. Of note, standard dose cyclophosphamide in the absence of an anthracycline does not seem to increase the risk of MDS/AML compared to the general population^202^.

It should, of course, be acknowledged that non-anthracycline regimens can also have distressing toxicity, such as neuropathy in the case of taxane/platinum regimens, and permanent alopecia which occurs in a dose-dependent fashion in a minority of patients with docetaxel, though these toxicities are not life-threatening.

While the absolute risk of anthracycline-related life-threatening toxicities (<5% for CHF, leukemia, and MDS) may seem inconsequential in the fight to avoid a metastatic recurrence of breast cancer, it is sobering to consider that for most patients, the absolute benefit expected to be imparted by adding an anthracycline to taxane-based therapy is similarly less than 5%.

## Conclusion

The following statement is true: as of this writing: there has been no prospective randomized trial that has demonstrated an OS benefit from the addition of anthracyclines to taxane-based chemotherapy in the curative setting. Although *HER2* amplification was thought to indicate a tumor subtype that would benefit from the addition of an anthracycline in an era that predated trastuzumab, no randomized study has shown the addition of anthracycline to a taxane/trastuzumab-based regimen improves outcomes for *HER2*-amplified breast cancer. While multiple markers have been postulated from retrospective analyses to identify those who will benefit from an anthracycline, most results were inconsistent and only one –*TOP2A*- has been evaluated prospectively. This marker may be the most biologically sound one tested as it is *the* target of the anthracyclines. Only one study has prospectively tested whether an anthracycline-based regimen adds benefit to *TOP2A* normal tumors and demonstrated no DFS or OS improvement^184^. In biomarker unselected patients with HER2- disease, a small absolute DFS benefit with the addition of an anthracycline to taxane-based therapy was noted in one study^109^, though several other trials did not confirm this benefit^62,110,111^. It is true that subset analyses of a handful of trials do suggest that for those patients with the heaviest disease burden (e.g., four or more nodes involved), the addition of an anthracycline to multiagent chemo regimens improves DFS^109,111^. However, in the era of mass breast cancer screening, fortunately, the majority of patients are not diagnosed with such locally advanced cancer. As we move into a future where we will likely be incorporating more biologically targeted therapies for those with the high-risk disease—a PARP inhibitor, olaparib, was just approved in the adjuvant setting for *BRCA*-mutation carriers and an adjuvant CDK4/6 inhibitor may indeed be available by the time this paper is published—the potential incremental benefits of adding an anthracycline will likely diminish further. Thus, as we select patients whose disease burden warrants the incorporation of an anthracycline into their regimen, we must also consider carefully that the potential life-altering toxicities associated with anthracyclines are real and are likely underreported. Thus, rather than asking which patients can be safely be treated *without* an anthracycline, we should be asking, does the data clearly exist to warrant the use of an anthracycline, keeping in mind that in many cases we are potentially harming patients more than helping them.

## Supplementary information

Supplementary Information

## Data Availability

Authors can confirm that all relevant data are included in the paper and/or its supplementary information files.
