# Supplementary material for: A careful reassessment of anthracycline use in curable breast cancer
Source: NPJ Breast Cancer. 2021 Oct 8;7:134. doi: 10.1038/s41523-021-00342-5 (PMC8501074; doi:10.1038/s41523-021-00342-5)
Supplement: Supplementary file 1 — Supplementary Information [file 41523_2021_342_MOESM1_ESM.pdf]

Supplementary Table 1a: Studies showing no benefit of anthracyclines (pre-taxanes)

Study (Year)	Ref	Regimens	N	Outcomes	P	Safety
NSABP-B12 (1989)<br>Fisher et al	23	PAF-Tam vs<br>PF-Tam	1106	5Y DFS: 64% vs 63%<br>5Y OS: 77% vs 78%	0.40<br>0.90	Symptomatic CHF: 0.6% vs 0%<br>Leukemia/MPD: 0.009% vs 0.007%
NSABP-B15 (1990)<br>Fisher et al	24	AC vs<br>AC-CMF vs<br>CMF	2194	3Y DFS: 62% vs 68% vs 63%<br>3Y DDFS: 68% vs 70% vs 68%<br>3Y OS: 83% vs 84% vs 82%	0.50<br>0.50<br>0.80	Symptomatic CHF: 0.1% vs 0% vs 0%<br>Leukemia/MPD: Not reported
Moliterni et al (1991)	25	CMF-A vs<br>CMF	486	5Y RFS: 72% vs 74%<br>5Y OS: 86% vs 89%	0.73<br>NR	Symptomatic CHF: 0% vs 0.004%<br>Leukemia/MPD: Not reported due to size
Naples GUN-3 (1995)<br>De Placido et al	26	CMF-EV vs<br>CMF	220	4Y DFS: 51% vs 46%<br>4Y OS: 77% vs 74%	0.66<br>0.58	Symptomatic CHF: None reported<br>Leukemia/MPD: None reported
Bang et al (2000)	27	AC vs<br>CMF	124	5Y DFS: 64% vs 78%<br>5Y OS: 90% vs 86%	0.12<br>0.96	Symptomatic CHF: None reported<br>Leukemia/MPD: None reported
Mauriac et al (1992)	28	MyTV-EVM vs<br>CMF	228	5Y Total RFS: 80% vs 69%<br>-Local RFS: 97% vs 94%<br>-Distant RFS: 84% vs 83%<br>5Y OS: No difference	0.05<br>NR<br>NR<br>NR	Symptomatic CHF: None reported<br>Leukemia/MPD: None reported
Budd et al (1995)	29	FAC-M vs<br>CMFVPd	531	5Y DFS: 50% vs 55%<br>5Y OS: 61% vs 64%	NR<br>0.06	Symptomatic CHF: 0.004% vs 0%<br>Leukemia: 0.007% vs 0%
Carpenter et al (1994)	30	CAF vs<br>CMF	528	5Y OS: 74% vs 68%	0.41	Symptomatic CHF: Unknown<br>Leukemia/MPD: Unknown
Levine et al (1990)	31	CMFVPd-ATam<br>(12 wks) vs<br>CMFVPd (36 wks)	437	3Y RFS: 55% vs 64%<br>3Y OS: 78% vs 85%	0.003<br>0.04	Symptomatic CHF: None reported<br>Leukemia: 0% vs 0.005%
Coombes et al (1996)	32	FEC vs<br>CMF	759	8Y RFS: 65% vs 64%<br>8Y OS: 82% vs 79%	0.61<br>0.13	Symptomatic CHF: 0.003% vs 0%<br>Leukemia: None reported
TAX 303<br>Piccart et al (2001)	33	EC vs<br>EC (high dose) vs<br>CMF	777	3Y EFS: 72% vs 80% vs 78%<br>3Y OS: 89% vs 92% vs 91%	NR*<br>NR*	Symptomatic CHF: 0.004% vs 0.01% vs 0%<br>Leukemia: 0% vs 0.01% vs 0%
NSABP-B23 (2001)<br>Fisher et al	34	AC+Tam vs<br>CMF+Tam**	2008	5Y RFS: 87% vs 87%<br>5Y EFS: 82% vs 83%<br>5Y OS: 90% vs 89%	0.96<br>0.80<br>0.80	Symptomatic CHF: None reported<br>Leukemia/MPD: None reported
INT-0102 (2005)<br>Hutchins et al	9	CAF vs<br>CMF	2690	10Y DFS: 77% vs 75%<br>10Y OS: 85% vs 82%	0.13<br>0.03†	Symptomatic CHF: Similar in both arms<br>Leukemia/MPD: Not increased in CAF arm

Supplementary Table 1b: Studies showing benefit of anthracyclines (pre-taxanes)

Study (Year)	Ref	Regimens	N	Outcomes (A v. NA)	P	Safety (A v. NA)
NSABP-B11 (1989)<br>Fisher et al	23	PAF<br>PF	707	5Y DFS: 51% vs 44%<br>5Y OS: 65% vs 59%	0.007<br>0.08	Symptomatic CHF: 0.6% vs 0.3%<br>Leukemia/MPD: 0% vs 0.003%
Misset et al (1996)	37	AVCF<br>CMF	249	16Y DFS‡: 53% vs 36%<br>16Y OS‡: 56% vs 41%	0.006<br>0.01	Symptomatic CHF: Similar in both arms<br>Leukemia/MDS: 0% vs 0%
Ejlertsen et al (2007)	38	CEF<br>CMF	1224	10Y DFS: 55% vs 50%<br>10Y OS: 62% vs 55%	0.04<br><0.01	Symptomatic CHF: Not increased in CEF<br>Leukemia/MPD: 0.003 vs 0.003
Poole et al (2006)	39	E-CMF<br>CMF	2401	5Y RFS: 76% vs 69%<br>5Y OS: 82% vs 75%	<0.001<br><0.001	Symptomatic CHF: None reported<br>Leukemia/MPD: 0.0008% vs 0%
Levine et al (1998)	35	CEF<br>CMF	710	10Y RFS: 52% vs 45%<br>10Y OS: 62% vs 58%	0.007<br>0.085	Symptomatic CHF: 1.1% vs 0.3%<br>Leukemia/MPD: 0.014% vs 0.003%
Martin et al (2003)	40	FAC<br>CMF	985	5Y DFS: 58% vs 50%<br>5Y OS: 75% vs 69%	0.056ƒ<br>NS ƒ	Cardiotoxicity: 2.1% vs 0.2%<br>Leukemia/MPD: None reported
De Placido et al (2005)	36	A-CMF<br>CMF	466	5Y DFS: 65% vs 54%<br>5Y OS: 83% vs 79%	0.044<br>0.26	Symptomatic CHF: None reported<br>Leukemia/MPD: None reported

Abbreviations:
P (melphalan), A (doxorubicin), F (fluorouracil), Tam (tamoxifen), C (cyclophosphamide), M (methotrexate), E (epirubicin), V (vincristine), My (mitomycin C), T (thiotepa), Pd (prednisone)
* - No difference in EFS or OS between CMF and EC (high dose) groups, but overall p value not reported for all three groups
** - study also looked at addition of tamoxifen vs placebo to each group, which did not impact any survival outcomes. Overall p values for all 4 groups are shown, but, for simplicity, survival percentages reported here represent tamoxifen including arms only.
† - slight benefit shown in OS in CAF group based on one-sided p value, but because of greater overall toxicity authors could not conclude CAF to be superior to CMF
‡ - median follow up 16 years (range 13-17 years)
ƒ – significant effect seen in prospectively formed node negative population, with (respectively for FAC vs CMF) 5Y DFS 75% vs 67%, P = 0.041; 5Y OS percentage not reported, but difference was significant (P = 0.034).
